# Digital credentials for environmental competencies: A review of open recognition systems in climate education

**DOI:** 10.12688/openreseurope.20048.1

**Published:** 2025-05-19

**Authors:** Pablo Martín-Ramos, Dileyni Diaz-De-Oleo, Fatma Fourati-Jamoussi, Kimberley Couchy, Lucio Alessandro Lo Giudice, Barbara Tosi, Frederico Oliveira-Pinto, Luís Veiga-Martins, Luis Manuel Navas-Gracia

**Affiliations:** 1 GIR-TADRUS, ETSIIAA, Universidad de Valladolid, Palencia, Castile and León, 34004, Spain; 2 InTerACT, UniLaSalle, Beauvais, Hauts-de-France, 60000, France; 3 Consorzio Scuola Comunità Impresa, Novara, NO, 28100, Italy; 4 NOVA School of Business and Economics, NOVA University, Lisbon, 2775-405, Portugal

**Keywords:** climate literacy, competency frameworks, educational innovation, environmental skills, lifelong learning, micro-credentials, open badges, education

## Abstract

Open education recognition systems are transforming how skills and competencies are validated across formal, informal, and non-formal learning environments. This literature review examines current practices in open recognition, with particular emphasis on micro-credentials, digital badges, and open educational resources (OER), while specifically exploring their application and potential in climate education. The analysis reveals significant potential for these systems to bridge academic learning with professional demands and environmental action, particularly through competency-based approaches. However, persistent challenges in standardization, technological integration, and stakeholder collaboration require attention for these systems to reach their full potential. This review aligns with the OpenPass4Climate (2022-1-FR01-KA220-HED-000089354) Erasmus+ project's goals of fostering sustainable, inclusive education systems by synthesizing key contributions, identifying challenges, and proposing recommendations from researchers in the field. Notable findings include the growing importance of micro-credentials for validating climate adaptation skills, the value of open badges in recognizing interdisciplinary competencies essential for environmental action, and the need for integrated qualification frameworks that can accommodate both formal and informal learning pathways in sustainability education. The review concludes by highlighting promising practices in Nordic regions where micro-credentials and open badges are being successfully employed in sustainable tourism education and climate adaptation learning networks.

## Plain language summary

This review examines how digital badges and micro-credentials can be used to recognize learning and actions related to climate change. These digital recognition tools provide ways to acknowledge skills and knowledge gained outside traditional education. We analyzed research published between 2010 and 2024 to understand current practices, challenges, and recommendations.

The review found that digital badges and micro-credentials can effectively validate climate-related competencies across various learning environments. They are particularly useful for recognizing interdisciplinary skills and action-oriented achievements essential for addressing climate challenges. However, there are significant obstacles to their widespread adoption, including lack of standardization, limited awareness among employers, and technical implementation barriers.

Based on the literature, we recommend integrating these recognition systems into institutional programs, aligning them with employer needs, developing clear design standards, and creating frameworks specifically for climate education. The findings suggest that with proper implementation, digital recognition systems could bridge formal education with practical environmental actions, offering flexible pathways for developing climate literacy and action competencies.

This research is part of the OpenPass4Climate Erasmus+ project, which aims to develop an open recognition system for climate-related achievements across European higher education institutions.

## Abstract (fr)

Les systèmes de reconnaissance de l'éducation ouverte transforment la façon dont les compétences sont validées dans les environnements d'apprentissage formels, informels et non formels. Cette revue de littérature examine les pratiques actuelles en matière de reconnaissance ouverte, en mettant particulièrement l'accent sur les micro-certifications, les badges numériques et les ressources éducatives libres (REL), tout en explorant spécifiquement leur application et leur potentiel dans l'éducation au climat. L'analyse révèle un potentiel significatif pour ces systèmes de faire le lien entre l'apprentissage académique, les exigences professionnelles et l'action environnementale, notamment grâce à des approches basées sur les compétences. Cependant, des défis persistants en matière de standardisation, d'intégration technologique et de collaboration entre les parties prenantes requièrent une attention particulière pour que ces systèmes atteignent leur plein potentiel. Cette revue s'aligne sur les objectifs du projet Erasmus+ OpenPass4Climate (2022-1-FR01-KA220-HED-000089354) visant à favoriser des systèmes éducatifs durables et inclusifs en synthétisant les contributions clés, en identifiant les défis et en proposant des recommandations issues des chercheurs du domaine. Les résultats notables incluent l'importance croissante des micro-certifications pour valider les compétences d'adaptation au climat, la valeur des badges ouverts dans la reconnaissance des compétences interdisciplinaires essentielles à l'action environnementale, et le besoin de cadres de qualification intégrés pouvant accueillir à la fois des parcours d'apprentissage formels et informels dans l'éducation au développement durable. La revue conclut en soulignant les pratiques prometteuses dans les régions nordiques où les micro-certifications et les badges ouverts sont utilisés avec succès dans l'éducation au tourisme durable et les réseaux d'apprentissage sur l'adaptation au climat.

## Abstract (de)

Offene Anerkennungssysteme im Bildungsbereich verändern die Art und Weise, wie Fähigkeiten und Kompetenzen in formellen, informellen und non-formalen Lernumgebungen validiert werden. Diese Literaturübersicht untersucht aktuelle Praktiken der offenen Anerkennung, mit besonderem Schwerpunkt auf Micro-Credentials, digitalen Abzeichen und offenen Bildungsressourcen (OER), während sie speziell deren Anwendung und Potenzial in der Klimabildung erforscht. Die Analyse zeigt ein erhebliches Potenzial dieser Systeme, akademisches Lernen mit beruflichen Anforderungen und Umweltmaßnahmen zu verbinden, insbesondere durch kompetenzbasierte Ansätze. Jedoch erfordern anhaltende Herausforderungen bei der Standardisierung, der technologischen Integration und der Zusammenarbeit der Interessengruppen Aufmerksamkeit, damit diese Systeme ihr volles Potenzial entfalten können. Diese Übersicht steht im Einklang mit den Zielen des Erasmus+-Projekts OpenPass4Climate (2022-1-FR01-KA220-HED-000089354), nachhaltige und inklusive Bildungssysteme zu fördern, indem sie wichtige Beiträge zusammenfasst, Herausforderungen identifiziert und Empfehlungen von Forschern auf diesem Gebiet vorschlägt. Zu den bemerkenswerten Ergebnissen gehören die wachsende Bedeutung von Micro-Credentials für die Validierung von Klimaanpassungsfähigkeiten, der Wert offener Abzeichen bei der Anerkennung interdisziplinärer Kompetenzen, die für Umweltmaßnahmen unerlässlich sind, und die Notwendigkeit integrierter Qualifikationsrahmen, die sowohl formelle als auch informelle Lernwege in der Nachhaltigkeitsbildung berücksichtigen können. Die Übersicht schließt mit der Hervorhebung vielversprechender Praktiken in nordischen Regionen, wo Micro-Credentials und offene Abzeichen erfolgreich in der Bildung für nachhaltigen Tourismus und in Lernetzwerken zur Klimaanpassung eingesetzt werden.

## Abstract (it)

I sistemi di riconoscimento dell'educazione aperta stanno trasformando il modo in cui competenze e abilità vengono validate negli ambienti di apprendimento formali, informali e non formali. Questa revisione della letteratura esamina le pratiche attuali nel riconoscimento aperto, con particolare enfasi su micro-credenziali, badge digitali e risorse educative aperte (OER), esplorando specificamente la loro applicazione e potenziale nell'educazione climatica. L'analisi rivela un potenziale significativo per questi sistemi di collegare l'apprendimento accademico con le esigenze professionali e l'azione ambientale, in particolare attraverso approcci basati sulle competenze. Tuttavia, sfide persistenti nella standardizzazione, nell'integrazione tecnologica e nella collaborazione tra le parti interessate richiedono attenzione affinché questi sistemi raggiungano il loro pieno potenziale. Questa revisione si allinea con gli obiettivi del progetto Erasmus+ OpenPass4Climate (2022-1-FR01-KA220-HED-000089354) di promuovere sistemi educativi sostenibili e inclusivi, sintetizzando contributi chiave, identificando sfide e proponendo raccomandazioni dai ricercatori del settore. I risultati notevoli includono la crescente importanza delle micro-credenziali per la validazione delle competenze di adattamento climatico, il valore dei badge aperti nel riconoscimento delle competenze interdisciplinari essenziali per l'azione ambientale, e la necessità di quadri di qualificazione integrati che possano accogliere sia percorsi di apprendimento formali che informali nell'educazione alla sostenibilità. La revisione si conclude evidenziando pratiche promettenti nelle regioni nordiche dove micro-credenziali e badge aperti vengono impiegati con successo nell'educazione al turismo sostenibile e nelle reti di apprendimento sull'adattamento climatico.

## Abstract (es)

Los sistemas de reconocimiento de la educación abierta están transformando la forma en que se validan las habilidades y competencias en entornos de aprendizaje formales, informales y no formales. Esta revisión de literatura examina las prácticas actuales en reconocimiento abierto, con particular énfasis en las microcredenciales, insignias digitales y recursos educativos abiertos (REA), al tiempo que explora específicamente su aplicación y potencial en la educación climática. El análisis revela un potencial significativo para que estos sistemas conecten el aprendizaje académico con las demandas profesionales y la acción ambiental, particularmente a través de enfoques basados en competencias. Sin embargo, los desafíos persistentes en estandarización, integración tecnológica y colaboración entre las partes interesadas requieren atención para que estos sistemas alcancen su pleno potencial. Esta revisión se alinea con los objetivos del proyecto Erasmus+ OpenPass4Climate (2022-1-FR01-KA220-HED-000089354) de fomentar sistemas educativos sostenibles e inclusivos mediante la síntesis de contribuciones clave, la identificación de desafíos y la propuesta de recomendaciones de investigadores en el campo. Los hallazgos notables incluyen la creciente importancia de las microcredenciales para validar habilidades de adaptación climática, el valor de las insignias abiertas en el reconocimiento de competencias interdisciplinarias esenciales para la acción ambiental, y la necesidad de marcos de cualificación integrados que puedan acomodar tanto vías de aprendizaje formales como informales en la educación para la sostenibilidad. La revisión concluye destacando prácticas prometedoras en las regiones nórdicas donde las microcredenciales y las insignias abiertas se están empleando con éxito en la educación turística sostenible y en redes de aprendizaje sobre adaptación climática.

## Introduction

### Overview of the OpenPass4Climate project

The global climate crisis demands not only awareness but meaningful commitment and action. The OpenPass4Climate (2022-1-FR01-KA220-HED-000089354) Erasmus+ project emerges as a response to this need. The project strives to transform climate-related educational initiatives, elevating them beyond awareness-building to foster concrete commitments to environmental action and climate justice. This transformation will be facilitated through the implementation of open recognition tools, specifically open badges integrated within the OpenPass4Climate digital platform. The project seeks to assess the tangible impact of these activities on driving real changes for climate justice and to understand the mechanisms that facilitate the rapid replication of positive behaviors in environmental contexts.

The implementation strategy encompasses a range of activities, from mapping eco-pedagogical initiatives to evaluating achievements through an Open Recognition System (ORS). The project actively advocates for a European vision, fostering an alliance of Young Achievers dedicated to climate causes. A pivotal aspect involves developing an OpenPass4Climate that is portable throughout one's life, designed to empower individuals from ages 10 to 80 to actively engage in climate-related endeavors and take pride in the badges they earn.

Anticipated outcomes include the establishment and growth of a community of climate achievers comprising staff, professors, researchers, and students. This community, acknowledged through the OpenPass4Climate platform without borders, is expected to expand beyond university boundaries to fulfill the aspirations of students seeking Open Badges for their ongoing contributions and to facilitate the acquisition of new knowledge while actively engaging in positive actions.

To effectively identify and evaluate diverse climate commitments, it is imperative to collect information concerning the nature of students' engagement with climate-related activities. This process encompasses both implicit and explicit forms of recognition. Completing this task requires a thorough understanding of current practices in open recognition systems and how they might be applied specifically to climate education.

### The rise of open recognition systems

The landscape of educational credentialing is undergoing significant transformation. While conventional academic credentials retain their significance, the educational landscape now embraces more nuanced and adaptable recognition mechanisms. These emerging approaches provide enhanced capacity to document and validate the multifaceted competencies developed across structured educational programs, community-based learning contexts, and self-directed knowledge acquisition pathways. Open badges and micro-credentials have emerged as prominent tools in this evolution, offering verification mechanisms for skills and knowledge that might otherwise go unrecognized ( West & Cheng, 2023).

Open recognition systems refer to flexible, technology-enabled frameworks—such as open badges and micro-credentials—that validate learning across formal, non-formal, and informal contexts. These systems enable learners to receive recognition for discrete skills and achievements, regardless of where or how the learning occurred.

While the terms "open badges" and "digital badges" are often used interchangeably, it is important to note their distinctions. "Open badges" specifically refer to a standardized format for digital credentials, such as those adhering to the Mozilla Open Badges standard, which ensures interoperability and portability across platforms. "Digital badges," on the other hand, is a broader term encompassing any digital representation of skills or achievements, which may or may not follow a specific standard. In the context of climate education, both open badges and broader digital badges offer promising solutions for recognizing interdisciplinary competencies and action-oriented achievements.

The concept of open recognition for learning achievements has gained significant traction. This development is particularly evident as educational pathways become more diverse. Additionally, the workforce increasingly demands more specific and demonstrable competencies. This trend has accelerated since the COVID-19 pandemic, which highlighted the need for flexible, accessible learning options and rapidly verifiable skills ( Tamoliune *et al.*, 2023). For climate education specifically, these systems offer the potential to recognize the multidisciplinary knowledge and action-oriented competencies that are essential for addressing complex environmental challenges.

### The relevance to climate education

Climate change education presents unique challenges that make it particularly suited to open recognition approaches. First, climate literacy spans multiple disciplines, from natural sciences to social studies, policy, economics, and beyond. Traditional educational structures often struggle to integrate this interdisciplinary knowledge effectively. Second, addressing climate change requires not just knowledge but action and civic engagement, which are rarely recognized in conventional academic credentials. Third, climate education happens across multiple contexts—formal classrooms, community initiatives, workplace projects, and personal advocacy—creating a need for recognition systems that can bridge these varied learning environments.

Open badges and micro-credentials offer promising solutions to these challenges. They can verify discrete competencies across disciplinary boundaries, recognize action-oriented achievements beyond knowledge acquisition, and validate learning regardless of where it occurs ( Cumberland *et al.*, 2023). This alignment between the needs of climate education and the affordances of open recognition systems suggests significant potential for their application in this domain.

### Purpose and scope of this review

This literature review aims to investigate current practices in open recognition systems, with a particular focus on their application and potential in climate education. It is conducted as part of Work Package 3 (WP3) of the OpenPass4Climate Erasmus+ project, which seeks to identify and evaluate different climate commitments and assess students' perceptions of their needs through an open recognition framework.

Specifically, this review addresses the following objectives:

– To examine the main contributions in the field of open recognition learning, particularly focusing on badges, micro-credentials, and their potential application in climate education contexts;
– To identify key challenges in implementing open recognition systems for climate-related competencies and actions; and
– To synthesize recommendations from existing research for effective implementation of open badges and micro-credentials in climate education.

By addressing these objectives, this review aims to provide a comprehensive foundation for the development of the OpenPass4Climate system, informing both the conceptual framework and practical implementation of open badges for recognizing climate action and learning.

## Method

### Research design

This review aims to identify, select, and analyze the existing literature on open recognition systems with a focus on their application to climate education. The methodology was designed to gather comprehensive evidence from diverse sources while remaining practical and focused on the specific needs of the OpenPass4Climate project.

### Search strategy and sources

***Databases***

To ensure comprehensive coverage of the literature, the following databases were searched:

– Web of Science
– EBSCOhost
– ERIC (Education Resources Information Center)
– Scopus
– Google Scholar
– Wiley
– PubMed/Europe PMC
– SemanticScholar

The selection of databases was deliberate and methodologically sound. Google Scholar was included for its comprehensive coverage across disciplines and ability to index a wide range of publication types, including those not captured by traditional academic databases. ERIC (Education Resources Information Center) was selected for its specialized focus on education literature, making it particularly valuable for identifying research on educational badges and credentials. Web of Science and Scopus were chosen for their rigorous indexing standards and citation tracking capabilities, facilitating the identification of highly cited, influential works in the field. The inclusion of EBSCOhost, Wiley, PubMed/Europe PMC, and SemanticScholar further ensured breadth of coverage across different publishers and disciplinary perspectives, which is particularly important given the interdisciplinary nature of climate education and open recognition systems

Additionally, we conducted searches in French, Portuguese, Italian, and German language sources to capture relevant literature from multiple linguistic contexts.

***Search terms and query development***

The search strategy was developed through an iterative process, focusing on the intersection of open recognition systems and climate or environmental education. The primary search strings used included:

*("open education recognition system" OR "open badges" OR "digital badges" OR "micro-credentials") AND ("climate change" OR "environmental actions" OR "ecopedagogical activities")*

Additional search strings were developed to refine results by educational context:

*("open education recognition system" OR "open badges" OR "digital badges") AND ("climate change" OR "environmental actions" OR "ecopedagogical activities") AND ("K-12" OR "higher education" OR "lifelong learning" OR "general public")*

Further search strings focused on methodological aspects, best practices, and international perspectives:

*("open education recognition system" OR "open badges" OR "digital badges") AND ("climate change" OR "environmental actions" OR "ecopedagogical activities") AND (methodology OR approach OR implementation)*
*("open education recognition system" OR "open badges" OR "digital badges") AND ("climate change" OR "environmental actions" OR "ecopedagogical activities") AND ("best practices" OR "successful cases" OR "exemplary models")*
*("open education recognition system" OR "open badges" OR "digital badges") AND ("climate change" OR "environmental actions" OR "ecopedagogical activities") AND ("international perspective" OR "global initiatives" OR "cross-cultural")*

These search strings were adapted as needed for each database and translated for non-English language searches. All search strings and results were documented to ensure transparency.

### Search results

Initial database searches yielded a significant number of records across all databases. After applying filters for publication date (2010–2024), peer-reviewed status, and removing duplicates, 477 unique records were identified for initial screening.

### Selection criteria and process

***Inclusion criteria***

Studies were included if they met the following criteria:

– Published between January 1, 2010, and November 30, 2024
– Published in peer-reviewed journals
– Available in full-text format (with university library membership)
– Focus on digital badges, open badges, or micro-credentials in educational or professional development contexts
– Keywords mentioned in the title or abstract

***Exclusion criteria***

Studies were excluded if they:

– Referred to digital badges when they were not considered as micro-credentials
– Lacked substantive discussion of open recognition systems or their application

***Screening and selection process***

The selection process consisted of an initial screening of titles and abstracts against the inclusion and exclusion criteria, followed by a full-text review of the remaining articles. This process resulted in a final sample of 69 articles, review papers, book chapters, and reports for analysis.

***Data analysis***

The analysis focused on identifying key themes, challenges, and recommendations related to open recognition systems for climate education.

### Limitations

Several limitations should be acknowledged:

– The review covers the literature published until January 31, 2025.
– Despite our multilingual approach, language barriers may have affected the comprehensiveness of non-English literature coverage.
– The keyword search requirements may have excluded relevant articles that addressed related concepts but used different terminology.

These limitations notwithstanding, this review provides a solid foundation for understanding the current state of research on open recognition systems for climate education within the context of the OpenPass4Climate project.

## Current practices in open recognition systems

The field of open learning recognition has seen significant contributions across various focus areas, each addressing different aspects of educational enhancement and validation of competencies. Across the studies reviewed, the main areas of open recognition learning can be grouped into key focus areas such as:

### Badges and micro-credentials

Digital badges and micro-credentials are increasingly vital for recognizing and validating skills across formal and informal learning contexts, particularly in climate education.

It is important to clearly distinguish between micro-credentials and open badges, as these terms are sometimes used interchangeably but represent distinct concepts. Micro-credentials are broader certification units that validate specific skills, knowledge, or competencies, typically requiring assessment and often carrying academic credit value. They represent smaller learning achievements than full degrees and may be stackable toward larger qualifications. Open badges, in contrast, are a specific technological implementation—a digital representation of skills, achievements, or qualities that contains verifiable metadata about the earner, issuer, criteria, and evidence. While all open badges could be considered micro-credentials, not all micro-credentials are implemented as open badges. The key distinction lies in their scope and technical implementation: micro-credentials focus on the certification framework and academic recognition, while open badges emphasize the digital, portable, and verifiable representation of achievements across platforms.

Oliver (2019) defines micro-credentials as certifications of assessed learning that complement or extend formal qualifications. West and Cheng (2023) emphasize their role in open education infrastructure, highlighting affordances such as granular recognition of accomplishments, mastery demonstration, and lifelong portfolio creation, which are especially relevant for documenting climate-related competencies. Similarly, Wolz *et al.* (2021) underscore their potential to digitize traditional graduation certificates, aligning with employers' digital recruitment processes and enhancing trust through secure, verifiable formats—attributes that could certify climate education achievements effectively.

A systematic review by Cumberland *et al.* (2023) found a growing interest in micro-credentials, particularly in higher education and workplace settings, where they can function either as pedagogical tools or as credentials that validate competencies. Digital badges represent a specific form of micro-credentialing that is gaining attention among employers and educational institutions alike.

Micro-credentials are seen as a means of certifying smaller attainments of learning than that of a full degree. This insight includes stackable credit, general recognition of prior learning, evidence of graduate attributes, and standards-based competencies associated with professional practice, promoting a structured approach to skills validation that bridges the gap between academic learning and professional demands ( Harris & Wihak, 2018). Authors assessing implementation and perceptions, such as Jirgensons and Kapenieks (2018) and Korhonen *et al.* (2020), found open badges useful for demonstrating competencies in job applications. However, Perkins and Pryor (2021) examined employer awareness and acceptance of digital badges, finding a significant gap in understanding that needs to be addressed for broader adoption. Dyjur and Lindstrom (2017) examined perceptions of digital badges, finding both positive and negative views. Davis and Singh (2015) identified badges as an opportunity to establish learner credibility outside of the original context but noted credibility as a dominant challenge requiring recognition by external audiences.

Micro-credentials demonstrate significant utility in domains outside traditional educational settings. Research conducted by Selvaratnam and Sankey (2021) identifies these compact certifications as offering Australian higher education institutions viable strategic directions for long-term sustainability, with particular effectiveness demonstrated in abbreviated learning modules and advanced graduate-level educational offerings. Similarly, a review by Varadarajan *et al.* (2023) suggests that micro-credentials can function as learning pathways, employment pathways, and qualification frameworks, serving the needs of different stakeholders including learners, educational institutions, employers, and governmental bodies.

According to Mah (2016), integrating micro-credentials into institutional programs can enhance their value and utility, making them more accessible and impactful for all stakeholders. Pollard and Vincent (2022) also proposed to align principles of micro-credentials with university missions and embed them into the curriculum. However, in this higher education context, Trepulé *et al.* (2021) emphasized the importance of clear evaluation and recognition criteria for digital badges to increase their value for assessment and recognition. In this line, the framework for the design and development of open badge systems proposed by Devedžić and Jovanović (2015) can be particularly helpful.

A recent perspective piece highlights how digital badges can offer self-guided learning options that allow individuals to develop both professionally and personally at their own pace. Currie *et al.* (2023) highlight how digital credentialing systems function as intrinsic motivation catalysts, stimulating investment in ongoing professional growth. These systems additionally create beneficial competitive dynamics that enhance learning outcomes, particularly when participation remains voluntary—a condition that promotes increased innovative thinking, deeper interaction with educational content, and heightened learner fulfillment.

Empirical research demonstrates the substantial motivational influence of open badge systems within recognition-based learning environments ( Abramovich *et al.*, 2013). Investigations by Cheng *et al.* (2018) reveal how digital credentialing mechanisms strengthen learners' self-regulation capabilities by supporting structured objective formulation, enhancing commitment to established targets, managing assignment difficulty levels, and integrating evaluative input. Research further suggests that shifting from traditional grading approaches to open badge frameworks redirects learner attention toward comprehensive skill acquisition and knowledge development ( Tomić *et al.*, 2024). Carey and Stefaniak (2018) found that skill-based badges are more valuable than participation badges.

In climate education, open badges provide a promising avenue for acknowledging interdisciplinary skills essential for environmental action. For instance, Rollin (2021) demonstrates their use in higher education to assess transversal skills like critical thinking and collaboration—key competencies for addressing climate challenges. This aligns with the findings by West and Cheng (2023) on how micro-credentials can support personalized learning pathways, motivating learners to develop sustainability skills through self-directed efforts.

Research by Miller and Jorre de St Jorre (2024) on employer perspectives in environmental science further validates the potential of micro-credentials in climate education. Through interviews with environmental professionals, they found that employers value alternative sources of evidence when recruiting graduates and showed enthusiasm for micro-credentials that could demonstrate specific environmental competencies, though they emphasized the need for rigorous standards and clear contextual information about these credentials.

Recent applications in Nordic coastal tourism education further demonstrate the practical value of micro-credentials and open badges in sustainability contexts. Halttunen *et al.* (2024) highlight how these credentials provide flexible, cost-effective pathways for tourism workers to develop sustainability competencies despite geographical constraints in remote destinations. Their research found that both tourism companies and destination management organizations recognized the complementary value of industry certificates and open badges for verifying skills in sustainability practices—with certificates meeting operational standards and badges demonstrating advanced expertise development. This application suggests that open badges could bridge formal education with practical environmental outcomes, enhancing learner engagement across diverse contexts, including the climate education field.

Additionally, Cox *et al.* (2020), cited in the ALN Climate Adaptation Micro-credential Strategy ( Cox, 2021), developed an openly licensed Climate Adaptation Competency Framework that aligns with micro-credential programs targeting professionals who need climate adaptation capabilities. Their framework identifies five competency domains with five sub-competencies each, structured to support criterion-based professional development in climate adaptation. This approach demonstrates how micro-credentials can be strategically designed to address workforce gaps in climate knowledge and skills, particularly in areas requiring strategic foresight for adaptation challenges.

### Open Educational Resources (OER) and Practices (OEP), MOOCs, and online learning

The adoption of open educational resources (OER) and open educational practices (OEP) has been widely explored, offering substantial benefits for enhancing learning accessibility and quality, particularly in climate education. West and Cheng (2023) argue that OER, when paired with open micro-credentials, forms a comprehensive open education infrastructure that supports personalized learning pathways—an approach that could democratize access to climate literacy.

Littlejohn and Hood (2017) demonstrated that OER engagement promotes three levels of learning for educators, which can be leveraged to structure workplace learning opportunities. van de Laar *et al.* (2024) found that open-access online courses were valued by Ph.D. students, although open micro-credentials did not incentivize course completion for this population. Bozkurt *et al.* (2019) and Knox (2013) highlighted the growing importance of openness in education, while also noticing challenges such as the divide between the global north and south in research output and the need for a critical exploration of the relationships between technology and education. Jung and Lee (2020) examined cultural factors influencing OER adoption and found that habit is a strong determinant across cultures, although other factors such as performance expectations and social influence also play a critical role. Bossu and Fountain (2015) introduced an open micro-course to build capacity in OEP. Adedoyin and Altinay (2023) examined factors that influence students' intentions to use OER. Knox (2013) proposed the inclusion of "open processes" in OEP. Ramirez-Montoya (2020) discussed how open education can support educational processes. Open science and scholarship promote transparency, accessibility, and collaboration in research. Corrall and Pinfield (2014) discussed the theoretical underpinnings of openness in different domains, emphasizing the importance of an integrated approach to open practices. Littlejohn and Hood (2017) studied how educators learn through engagement with OER, demonstrating the transformative potential of open educational resources in professional development. Askeroth and Newby (2020) noted that badges are well-suited for adult learners in less formal educational settings.

Massive open online courses (MOOCs) have been instrumental in providing scalable and flexible learning opportunities. The most significant contribution came from Ramirez-Montoya *et al.* (2022), who identified key factors influencing MOOC effectiveness, such as perceived usefulness, self-efficacy, and achievement motivation, which are critical to designing effective online learning experiences. Gates and Walters (2015) explored the relevance of MOOCs in social work education, highlighting their potential in delivering professional training. Kopp and Ebner (2017) found that the awarding of certificates has an impact on MOOC learners, but the impact varies depending on the target group, commitment, and usability. Other studies have contributed by examining approaches to assessment, such as Farrow *et al.* (2021), who proposed identity verification strategies for MOOC platforms, and Chauhan (2014), who discussed automated assessment techniques in MOOCs. "Smart systems" are being developed to track and predict learner behavior. Bossu and Fountain (2015) acknowledge that Applaud —a specific online scheme system to gain recognition— has had a positive impact on learning and teaching at the Open University, supporting professional development, career development, and reflective practice. Charleer *et al.* (2013) found that gamified dashboards such as Navi Badgeboard helped with awareness of personal activities, while Navi Surface improved collaboration and reflection.

Further applications of OER and MOOCs in climate education underscore their synergy with open recognition systems. Cieply and Grand (2019) analyzed the use of open badges in higher education to recognize competencies developed through OER-based learning, offering a scalable model for climate education that fosters critical engagement with ecological issues. This complements the emphasis of West and Cheng (2023) on micro-credentials supporting inquiry-based learning, which could enhance climate-focused curricula across educational levels.

### Recognition of prior and non-formal learning

Regarding the recognition of prior and non-formal learning, Schmidt *et al.* (2009) proposed a peer-based method of assessment and recognition that uses online communities to provide a credible alternative to traditional assessment methods. Tereseviciene *et al.* (2020) studied the recognition of open online learning in higher education and identified challenges in adapting to changes in the labor market. Friesen and Wihak (2013) explored the links between open educational contexts and prior learning assessment and recognition (PLAR). Harris and Wihak (2018) identified synergies in the recognition of non-formal learning. The integration of technology in higher education is essential to adapt to the changing demands of the workforce. Therefore, Lang (2023) argued that universities should adopt digital micro-credentials to meet the changing needs of the workforce and facilitate a shift toward more flexible and relevant educational offerings. Orr *et al.* (2019) identified archetypes of technology-enabled higher education, providing a framework for understanding different levels of digital adoption in institutions. Volungevičienė *et al.* (2020) supported the shift to learner-centered, multidimensional teaching models that leverage new digital devices to create more engaging and effective learning environments. Zawacki-Richter *et al.* (2020) provided a comprehensive review of open and distance learning research, identifying trends and gaps in the current landscape.

The COVID-19 pandemic has accelerated the adoption of distance education across all types of schools, creating both opportunities and challenges for Open Universities ( Lyu, 2023). While this expansion has made micro-credentials more relevant, it also raises concerns about how to effectively implement online teaching and maintain educational quality. The pandemic has stimulated the need for developing new competencies and personalized, competency-based, flexible, and collaborative professional development ( Varadarajan *et al.*, 2023). These changes have highlighted the importance of micro-credentials as tools for rapid upskilling in an educational landscape that increasingly embraces online delivery modalities.

Another contribution of reviewed research on open learning is the recognition of competency frameworks and sustainability in education, which are critical to aligning skills with evolving industry needs and promoting lifelong learning. Fitsilis (2024) emphasized the importance of competency frameworks in Education 4.0 that align individual skills with organizational goals. Annelin and Boström (2022) analyzed questionnaire assessment tools for measuring sustainability competencies and identified the need for comprehensive assessment tools that span multiple disciplines.

## Challenges

### Standardization and definitional issues

The lack of standardized definitions and criteria for openness leads to inconsistent results. Different interpretations of what constitutes open learning recognition can lead to differences in implementation and effectiveness, as noted in several studies.

A persistent challenge in the micro-credential landscape is the absence of global standardization, complicating their application in climate education. For instance, a climate adaptation course might be valued differently across regions due to varying credit definitions, hindering learners’ ability to transfer credentials internationally. Ahsan *et al.* (2023) note the lack of consensus on micro-credential definitions and credit values across universities, industries, and governments, a concern echoed by Wolz *et al.* (2021), who identify terminological ambiguity in higher education institutions as a barrier to adoption. For example, the European Credit Transfer System defines micro-credentials as conferring a minimum of 5 ECTS credits, while the New Zealand Qualifications Authority bounds them between 5 and 40 credits ( Varadarajan *et al.*, 2023).

### Institutional coherence and coordination

One of the challenges reported in the reviewed studies was the lack of coherence and coordination between different open initiatives. It has been highlighted this issue of the unified policy approach to integrate different open domains to maximize their potential benefits for learners ( Corrall & Pinfield, 2014). It has also been mentioned as a limitation in the widespread adoption and implementation of open learning recognition tools that it requires significant cultural change within educational institutions.

### Stakeholder awareness and acceptance

Perkins and Pryor (2021) found a significant gap in employer awareness and acceptance of digital badges, indicating the need for broader educational efforts to increase understanding and acceptance in formal and informal learning environments. Miller and Jorre de St Jorre (2024) further confirmed this challenge in environmental science contexts, where employers expressed support for micro-credentials but required more context about their application and assurance of their rigor.

The research by Halttunen *et al.* (2024) additionally revealed unique challenges in implementing open badges for sustainability education in tourism, particularly in remote or seasonal destinations where access to training is limited by geographical and economic constraints. Their findings suggest that while digital credentials can overcome some of these barriers, they must be designed with awareness of contextual limitations like connectivity issues or variable workforce composition throughout the year.

Cumberland *et al.* (2023) conducted a systematic review of the digital badge landscape in educational and workplace settings, identifying two main uses: as pedagogical tools and as micro-credentials. Their work identifies persistent confusion in nomenclature and a divergence in findings that suggests the need for further exploration. The authors note that for digital badges to be valuable, employers must recognize them as such, which presents both a challenge and an opportunity for collaboration between educational institutions and the labor market.

### Technological and implementation barriers

In addition, technological challenges such as scalability, privacy, and storage capacity, particularly with blockchain technology, pose significant barriers. Jirgensons and Kapenieks (2018) discuss these issues in the context of implementing blockchain for digital learning. Korhonen *et al.* (2019) reported that learners attached earned open badges to their ePortfolios and shared them in digital environments to showcase their skills. Organizational barriers, including resistance from traditional academic and publishing sectors, also hinder the adoption of open practices. In addition to technological challenges, sharing research data and educational resources often requires additional effort, which can be a restraint for educators and researchers. The additional workload and lack of support mechanisms are significant barriers to wider adoption.

Research by Ahsan *et al.* (2023) also identified technological readiness as a critical factor in micro-credential implementation. Without supportive learning technology, implementing micro-credentials in higher education becomes impractical. The authors noted that blockchain technology could potentially enhance the authenticity of micro-credentials through certified digital documents, though further research on its adoption is needed. Wolz *et al.* (2021) discuss blockchain's potential to enhance credential security, yet note scalability and privacy challenges that could hinder its use in certifying environmental achievements. A French report by Rollin (2022) identifies methodological issues—reliability, validity, and equity—in assessing transversal skills via open badges, underscoring the need for robust frameworks to ensure equitable recognition of climate education outcomes across diverse learners.

### Climate education specific challenges

The Climate Adaptation Micro-credential Strategy ( Cox, 2021) also points to the challenge of creating comprehensive recognition systems that can effectively connect disparate educational offerings related to climate adaptation. The strategy highlights the need for constant interaction between didactics and professional practice to ensure that micro-credentials meet real-world needs in addressing climate challenges. This connects to broader concerns about fragmentation in sustainability education, where many initiatives exist but often lack coherent frameworks that allow for progressive skill-building across different contexts.

## Recommendations

The literature review highlights several key recommendations for the effective implementation of micro-credentials and digital badges. These include integrating them into institutional programs to ensure they add value and are easy to use with clear metrics; embedding them in the curriculum using critical and reflective pedagogy ( Mah, 2016; Pollard & Vincent, 2022); and making digital credentials easy to understand and valuable to employers and also aligning pedagogical practices with employer needs ( Perkins & Pryor, 2021). McNamara and Sun (2021) called for a global agreement on standards for the recognition, creation, validation, and distribution of micro-credentials and digital badges.

### Institutional integration and policy

The systematic analysis of digital credentialing practices by Cumberland *et al.* (2023) recommends that tertiary education organizations develop governance frameworks and institutional regulations prior to implementation. Their findings emphasize the necessity of creating structured classification systems that delineate how micro-credentials integrate within institution-specific educational frameworks. Their research identifies concentrated learning experiences and advanced degree programming as optimal starting points for micro-credential integration due to their reduced implementation risks. The authors highlight the importance of standardization to ensure portability between institutions, an aspect that has also been pointed out by other recent research, including McNamara and Sun (2021).

### Employer and stakeholder alignment

Recent reviews indicate that employers value micro-credentials as a way to fulfill their specific requirements and acknowledge skills using digital badges ( Varadarajan *et al.*, 2023). However, employers express concerns about the consistency of micro-credentials, their integrity, and the potential for fraudulent credentials. As noted by Kahle-Piasecki *et al.* (2024), the success of micro-credentials in the industry depends on standardization and recognition, suggesting the need for partnerships between higher education institutions, industry, and government agencies. This aligns with Perkins and Pryor (2021), who stress making credentials valuable and understandable to employers.

### Technical and design aspects

*Badge system design*. Develop badge systems that provide visible recognition with metadata linked to validating evidence of educational achievement ( Gibson *et al.*, 2023; Gibson *et al.*, 2015). Design badge systems that encourage teamwork and public display of achievement ( Goodyear & Nathan-Roberts, 2017).

*Technical ecosystem*. Sousa-Vieira *et al.* (2021) suggest that the successful implementation of digital badges in higher education requires a comprehensive technical ecosystem. This includes considerations for storage, security, and administrative management of badges to maintain their integrity and authenticity. According to their conceptual framework, the badge module should include properties such as name, description, icon, text, visibility settings, and type (automatic, manual, or by contest).

*Blockchain solutions*. Wolz *et al.* (2021) propose blockchain-based systems to secure digital credentials, offering a scalable solution for documenting lifelong climate learning—a strategy that could build trust among EU stakeholders.

### Pedagogical approaches

Werth and Williams (2022) advocate building on open pedagogy to democratize learning experiences. McDaniel (2016) suggested the use of a design matrix and design recommendations for badges derived from diverse perspectives. Addressing cultural and contextual differences is essential to effectively adopt open learning recognition tools. Additionally, Mah (2016) and Pollard and Vincent (2022) emphasize embedding micro-credentials in the curriculum using critical and reflective pedagogy to enhance educational value.

### Applications in climate education

*Environmental science context*. Based on their research in environmental science fields, Miller and Jorre de St Jorre (2024) recommend that micro-credential developers provide clear context about how these credentials can be used in professional settings and ensure rigorous assessment standards to build employer confidence. Their findings suggest that environmental employers value multiple lines of evidence when recruiting, and micro-credentials could serve as valuable supplementary validation of skills if properly contextualized.

*Nordic tourism education*. In the context of Nordic tourism education, Halttunen *et al.* (2024) suggest a blended learning approach that combines on-site workplace training with digital badges and MOOCs to accommodate learning in remote destinations. Their research emphasizes the importance of establishing common proficiency goals as frames of reference for sustainability competencies and providing flexible recognition mechanisms that can validate both existing and newly developed skills. This approach could be particularly relevant for climate education in contexts where geographical barriers limit access to traditional educational opportunities.

*Climate adaptation strategy*. The Climate Adaptation Micro-credential Strategy ( Cox, 2021) proposes a strategic approach for climate education that involves basing micro-credentials on open competency frameworks that clearly articulate required skills across different domains of climate adaptation work. Their recommendations include:

–    Certifying courses using established competency frameworks to provide clear standards for employers and learners

–    Designing micro-credentials for hybrid delivery with both synchronous and asynchronous components to accommodate working professionals

–    Awarding credentials digitally with verifiable critical information summaries that provide metadata about the competencies achieved

–    Creating pathways for prior learning assessment that allow experienced professionals to demonstrate existing climate adaptation skills

These recommendations are particularly relevant for developing open recognition systems in climate education as they address both pedagogical design and practical implementation concerns.

*Accessibility and learner agency*. To advance open recognition in climate education, both accessibility and learner agency should be prioritized. Jean-Marie and Millet (2022) advocate making badge tools accessible to all educational actors, ensuring equitable participation in climate-focused programs.

### Future research directions

Further research and development are essential to advance the recognition of open learning. For instance, Jirgensons and Kapenieks (2018) suggest conducting case studies to explore the potential of blockchain technology in different courses and programs. Jung and Lee (2020) suggested conducting qualitative or mixed methods studies to understand educators' use of OER within and across cultures. Lee (2020) recommended focusing efforts on opening higher education to disadvantaged groups and collecting real-life stories to inform practice. Randall *et al.* (2019) recommended further research to support the use of instructional design assistants for badge creation, as innovative pedagogical approaches and thoughtful design are key to successful implementation. The scholarly discourse surrounding recognition systems continues to evolve, with Roy and Clark (2019) particularly emphasizing the necessity for expanded investigation across varied educational and cultural environments. Contemporary research affirms the enduring relevance of digital credentialing mechanisms within educational innovation ( Farmer & West, 2016), yet significant knowledge gaps persist that require targeted investigation to advance understanding of their full potential and limitations.

## Conclusion

Research on the recognition of open learning, including digital badges, micro-credentials, and OER underscores their potential to transform education and professional development, particularly in climate-related fields. However, realizing this potential requires overcoming significant challenges related to standardization, credibility, technological barriers, and integration with existing systems.

The literature highlights the need for stronger partnerships between higher education and employers to establish effective digital credentialing systems. The recommendations provided by researchers offer a roadmap for integrating these tools into education systems, ensuring quality and relevance, and fostering a culture of openness and inclusiveness. Continued research and collaboration among stakeholders are essential to advance the field and fully realize the benefits of open learning credentials.

This review demonstrates the multifaceted nature of research on open credentialing, including aspects of design, psychology, credibility, educational outcomes, specific applications, engagement factors, and stakeholder perceptions. Key contributions include: (1) documenting the rise of micro-credentials and open badges in validating climate competencies, (2) identifying standardization and employer awareness as critical challenges, and (3) synthesizing actionable recommendations for climate education integration. Future research should focus on developing best practices for badge design, exploring their long-term impact on learning outcomes, and investigating ways to increase their recognition and value in the labor market, particularly in climate education contexts.

The emerging applications in Nordic regions and climate adaptation learning networks suggest promising directions for open recognition systems in climate education. These approaches emphasize the importance of contextualized, competency-based frameworks that can accommodate both formal and informal learning pathways while addressing the practical challenges of climate adaptation work.

Recent systematic reviews emphasize the need for an integrative approach to micro-credential implementation that considers the perspectives of all stakeholders—learners, higher education institutions, employers, and governmental bodies. The success of micro-credentialing systems will depend on addressing challenges related to standardization, technological readiness, and stakeholder perceptions while maximizing opportunities for flexible, personalized learning pathways and enhanced employability in climate-related fields.

## Ethics and consent

Ethical approval and consent were not required.

## Data Availability

No data are associated with this article.
