# Digital credentials for environmental competencies: A scoping review of open recognition systems in climate education

**DOI:** 10.12688/openreseurope.20048.4

**Published:** 2026-02-11

**Authors:** Pablo Martín-Ramos, Dileyni Diaz-De-Oleo, Fatma Fourati-Jamoussi, Kimberley Couchy, Lucio Alessandro Lo Giudice, Barbara Tosi, Frederico Oliveira-Pinto, Luís Veiga-Martins, Luis Manuel Navas-Gracia

**Affiliations:** 1 GIR-TADRUS, ETSIIAA, Universidad de Valladolid, Palencia, Castile and León, 34004, Spain; 2 InTerACT, UniLaSalle, Beauvais, Hauts-de-France, 60000, France; 3 Consorzio Scuola Comunità Impresa, Novara, NO, 28100, Italy; 4 Reitoria da Universidade Nova de Lisboa, Campus de Campolide, Lisboa, 1099-085, Portugal

**Keywords:** climate literacy, competency frameworks, educational innovation, environmental skills, lifelong learning, micro-credentials, open badges, education

## Abstract

Open education recognition systems are transforming how skills and competencies are validated across formal, informal, and non-formal learning environments. This scoping review examines current practices in open recognition systems—including micro-credentials, digital badges, and open educational resources (OER) completion certificates and participation badges—with particular emphasis on their application and potential in climate education. Following systematic search strategies across eight databases, we analyzed 70 publications (2010–2024) comprising journal articles, book chapters, institutional reports, and conference proceedings to map existing evidence, identify key concepts, and uncover gaps in applying these systems to climate education. The analysis reveals that while these systems have significant potential to bridge academic learning with professional demands and environmental action, their impact is limited by persistent challenges: lack of definitional consistency and quality-assurance standards across institutions; significant technological barriers to credential portability and verification; and lack of awareness and trust from employers, undermining these credentials’ currency in the labor market. Despite these challenges, notable findings include the growing importance of micro-credentials for validating climate adaptation skills, the value of open badges in recognizing interdisciplinary competencies essential for environmental action, and the need for integrated qualification frameworks that can accommodate both formal and informal learning pathways in sustainability education. This integration is critical for validating the interdisciplinary and action-oriented skills demanded by the green economy, often acquired through the non-formal and informal learning pathways characteristic of environmental action and advocacy. Our review concludes that a significant implementation and research gap exists between open recognition’s potential and its application in climate education. Most notably, we find a near-complete absence of recognition frameworks for eco-pedagogical activities, perpetuating focus on technical knowledge over transformative, action-oriented learning. We recommend that future work prioritize creating robust, co-designed assessment models for these critical competencies to bridge the gap between education and meaningful climate action.

## Plain language summary

This review examines how digital badges and micro-credentials can be used to recognize learning and actions related to climate change. These digital recognition tools provide ways to acknowledge skills and knowledge gained outside traditional education. We analyzed research published between 2010 and 2024 to understand current practices, challenges, and recommendations.

The review found that digital badges and micro-credentials can effectively validate climate-related competencies across various learning environments. They are particularly useful for recognizing interdisciplinary skills and action-oriented achievements essential for addressing climate challenges. However, there are significant obstacles to their widespread adoption, including lack of standardization, limited awareness among employers, and technical implementation barriers.

Based on the literature, we recommend integrating these recognition systems into institutional programs, aligning them with employer needs, developing clear design standards, and creating frameworks specifically for climate education. The findings suggest that with proper implementation, digital recognition systems could bridge formal education with practical environmental actions, offering flexible pathways for developing climate literacy and action competencies.

This research is part of the OpenPass4Climate Erasmus+ project, which aims to develop an open recognition system for climate-related achievements across European higher education institutions.

## Abstract (fr)

Les systèmes de reconnaissance de l’éducation ouverte transforment la validation des compétences dans les environnements d’apprentissage formels, informels et non formels. Cette revue exploratoire examine les pratiques actuelles des systèmes de reconnaissance ouverte—incluant micro-certifications, badges numériques, et certificats de completion et badges de participation aux ressources éducatives libres (REL)—en mettant l’accent sur leur application et potentiel dans l’éducation climatique. Suivant des stratégies de recherche systématique dans huit bases de données, nous avons analysé 70 publications (2010-2024) comprenant articles de journaux, chapitres de livres, rapports institutionnels et actes de conférences pour cartographier les preuves existantes, identifier les concepts clés et découvrir les lacunes dans l’application de ces systèmes à l’éducation climatique. L’analyse révèle que bien que ces systèmes aient un potentiel significatif pour relier l’apprentissage académique aux demandes professionnelles et à l’action environnementale, leur impact est limité par des défis persistants : manque de cohérence définitionnelle et de normes d’assurance qualité entre institutions ; barrières technologiques importantes à la portabilité et vérification des certifications ; et manque critique de sensibilisation et confiance des employeurs, compromettant la valeur de ces certifications sur le marché du travail. Malgré ces défis, les résultats notables incluent l’importance croissante des micro-certifications pour valider les compétences d’adaptation climatique, la valeur des badges ouverts pour reconnaître les compétences interdisciplinaires essentielles à l’action environnementale, et le besoin de cadres de qualification intégrés accommodant les parcours d’apprentissage formels et informels dans l’éducation à la durabilité. Notre revue conclut qu’un écart significatif existe entre le potentiel de la reconnaissance ouverte et son application dans l’éducation climatique. Nous trouvons une absence quasi-complète de cadres de reconnaissance pour les activités écopédagogiques, perpétuant la focalisation sur la connaissance technique plutôt que l’apprentissage transformateur orienté vers l’action

## Abstract (de)

Offene Bildungsanerkennungssysteme transformieren die Validierung von Fähigkeiten und Kompetenzen in formellen, informellen und non-formalen Lernumgebungen. Diese Scoping-Übersicht untersucht aktuelle Praktiken in offenen Anerkennungssystemen—einschließlich Mikro-Zertifikaten, digitalen Abzeichen und Abschlusszertifikaten sowie Teilnahmeabzeichen für offene Bildungsressourcen (OER)—mit besonderem Schwerpunkt auf Anwendung und Potenzial in der Klimabildung. Durch systematische Suchstrategien in acht Datenbanken analysierten wir 70 Publikationen (2010-2024) bestehend aus Zeitschriftenartikeln, Buchkapiteln, institutionellen Berichten und Konferenzbeiträgen, um vorhandene Belege zu kartieren, Schlüsselkonzepte zu identifizieren und Lücken in der Anwendung auf Klimabildung aufzudecken. Die Analyse zeigt, dass diese Systeme erhebliches Potenzial haben, akademisches Lernen mit beruflichen Anforderungen und Umweltmaßnahmen zu verbinden, ihre Wirkung jedoch durch anhaltende Herausforderungen begrenzt ist: Mangel an definitorischer Konsistenz und Qualitätssicherungsstandards zwischen Institutionen; erhebliche technologische Barrieren für Portabilität und Verifizierung von Zertifikaten; und kritisch, Mangel an Bewusstsein und Vertrauen seitens der Arbeitgeber, was die Währung dieser Zertifikate auf dem Arbeitsmarkt untergräbt. Trotz dieser Herausforderungen umfassen bemerkenswerte Erkenntnisse die wachsende Bedeutung von Mikro-Zertifikaten für die Validierung von Klimaanpassungsfähigkeiten, den Wert offener Abzeichen bei der Anerkennung interdisziplinärer Kompetenzen für Umweltmaßnahmen und die Notwendigkeit integrierter Qualifikationsrahmen für formelle und informelle Lernwege in der Nachhaltigkeitsbildung. Unsere Übersicht schließt, dass eine erhebliche Implementations- und Forschungslücke zwischen dem Potenzial offener Anerkennung und ihrer Anwendung in der Klimabildung besteht. Wir finden eine nahezu vollständige Abwesenheit von Anerkennungsrahmen für öko-pädagogische Aktivitäten, was eine Fokussierung auf technisches Wissen über transformatives, handlungsorientiertes Lernen perpetuiert.

## Abstract (it)

I sistemi di riconoscimento dell’educazione aperta stanno trasformando la validazione di competenze negli ambienti di apprendimento formali, informali e non formali. Questa revisione esplorativa esamina le pratiche attuali nei sistemi di riconoscimento aperto—inclusi micro-credenziali, badge digitali, e certificati di completamento e badge di partecipazione delle risorse educative aperte (OER)—con particolare enfasi su applicazione e potenziale nell’educazione climatica. Seguendo strategie di ricerca sistematiche attraverso otto database, abbiamo analizzato 70 pubblicazioni (2010-2024) comprendenti articoli di riviste, capitoli di libri, rapporti istituzionali e atti di conferenze per mappare le evidenze esistenti, identificare concetti chiave e scoprire lacune nell’applicazione di questi sistemi all’educazione climatica. L’analisi rivela che mentre questi sistemi hanno potenziale significativo per collegare l’apprendimento accademico con richieste professionali e azione ambientale, il loro impatto è limitato da sfide persistenti: mancanza di coerenza definitoria e standard di assicurazione qualità tra istituzioni; barriere tecnologiche significative alla portabilità e verifica delle credenziali; e criticamente, mancanza di consapevolezza e fiducia da parte dei datori di lavoro, minando la valuta di queste credenziali nel mercato del lavoro. Nonostante queste sfide, i risultati notevoli includono la crescente importanza delle micro-credenziali per validare competenze di adattamento climatico, il valore dei badge aperti nel riconoscere competenze interdisciplinari essenziali per l’azione ambientale, e necessità di quadri di qualificazione integrati che accommodino percorsi di apprendimento formali e informali nell’educazione alla sostenibilità. La nostra revisione conclude che esiste un gap significativo di implementazione e ricerca tra il potenziale del riconoscimento aperto e la sua applicazione nell’educazione climatica. Troviamo un’assenza quasi completa di quadri di riconoscimento per attività ecopedagogiche, perpetuando focus sulla conoscenza tecnica piuttosto che apprendimento trasformativo orientato all’azione.

Los sistemas de reconocimiento de la educación abierta están transformando la validación de habilidades y competencias en entornos de aprendizaje formales, informales y no formales. Esta revisión exploratoria examina las prácticas actuales en sistemas de reconocimiento abierto—incluyendo microcredenciales, insignias digitales, y certificados de finalización e insignias de participación de recursos educativos abiertos (REA)—con particular énfasis en su aplicación y potencial en la educación climática. Siguiendo estrategias de búsqueda sistemática a través de ocho bases de datos, analizamos 70 publicaciones (2010-2024) que comprenden artículos de revistas, capítulos de libros, informes institucionales y actas de conferencias para mapear la evidencia existente, identificar conceptos clave y descubrir brechas en la aplicación de estos sistemas a la educación climática. El análisis revela que aunque estos sistemas tienen potencial significativo para conectar el aprendizaje académico con demandas profesionales y acción ambiental, su impacto está limitado por desafíos persistentes: falta de consistencia definitoria y estándares de aseguramiento de calidad entre instituciones; barreras tecnológicas significativas para la portabilidad y verificación de credenciales; y críticamente, falta de conciencia y confianza por parte de empleadores, socavando la moneda de estas credenciales en el mercado laboral. A pesar de estos desafíos, los hallazgos notables incluyen la creciente importancia de las microcredenciales para validar habilidades de adaptación climática, el valor de las insignias abiertas en reconocer competencias interdisciplinarias esenciales para la acción ambiental, y necesidad de marcos de calificación integrados que acomoden vías de aprendizaje formales e informales en la educación para la sostenibilidad. Nuestra revisión concluye que existe una brecha significativa de implementación e investigación entre el potencial del reconocimiento abierto y su aplicación en la educación climática. Encontramos una ausencia casi completa de marcos de reconocimiento para actividades ecopedagógicas, perpetuando enfoque en conocimiento técnico sobre aprendizaje transformativo orientado a la acción.

## Abstract (es)

Los sistemas de reconocimiento de la educación abierta están transformando la forma en que se validan las habilidades y competencias en entornos de aprendizaje formales, informales y no formales. Esta revisión de literatura examina las prácticas actuales en reconocimiento abierto, con particular énfasis en las microcredenciales, insignias digitales y recursos educativos abiertos (REA), al tiempo que explora específicamente su aplicación y potencial en la educación climática. El análisis revela un potencial significativo para que estos sistemas conecten el aprendizaje académico con las demandas profesionales y la acción ambiental, particularmente a través de enfoques basados en competencias. Sin embargo, los desafíos persistentes en estandarización, integración tecnológica y colaboración entre las partes interesadas requieren atención para que estos sistemas alcancen su pleno potencial. Esta revisión se alinea con los objetivos del proyecto Erasmus+ OpenPass4Climate (2022-1-FR01-KA220-HED-000089354) de fomentar sistemas educativos sostenibles e inclusivos mediante la síntesis de contribuciones clave, la identificación de desafíos y la propuesta de recomendaciones de investigadores en el campo. Los hallazgos notables incluyen la creciente importancia de las microcredenciales para validar habilidades de adaptación climática, el valor de las insignias abiertas en el reconocimiento de competencias interdisciplinarias esenciales para la acción ambiental, y la necesidad de marcos de cualificación integrados que puedan acomodar tanto vías de aprendizaje formales como informales en la educación para la sostenibilidad. La revisión concluye destacando prácticas prometedoras en las regiones nórdicas donde las microcredenciales y las insignias abiertas se están empleando con éxito en la educación turística sostenible y en redes de aprendizaje sobre adaptación climática.

## Introduction

The global climate crisis demands educational approaches that move beyond awareness-raising to cultivate competencies, values, and forms of engagement that support sustainable societal transitions. Simultaneously, higher education institutions, professional development providers, and community initiatives are increasingly experimenting with digital badges, micro-credentials, and other open recognition systems to validate learning occurring across formal, non-formal, and informal contexts. Despite this parallel evolution, the intersection of open recognition systems and climate education remains underexplored. This scoping review addresses that gap by examining how open recognition systems are currently conceptualized and implemented, and to what extent they support climate-related learning, environmental action, and eco-pedagogical practices.

### Overview of the OpenPass4Climate project

The OpenPass4Climate (2022-1-FR01-KA220-HED-000089354) Erasmus+ project responds directly to these challenges. The project strives to transform climate-related educational initiatives, elevating them beyond awareness-building to foster concrete commitments to environmental action and climate justice. This transformation will be facilitated through the implementation of open recognition tools, specifically open badges integrated within the OpenPass4Climate digital platform. The project seeks to assess the tangible impact of these activities on driving real changes for climate justice and to understand the mechanisms that facilitate the rapid replication of positive behaviors in environmental contexts.

The implementation strategy encompasses a range of activities, from mapping eco-pedagogical initiatives to evaluating achievements through an Open Recognition System (ORS). The project actively advocates for a European vision, fostering an alliance of Young Achievers dedicated to climate causes. A pivotal aspect involves developing an OpenPass4Climate that is portable throughout one’s life, designed to empower individuals from ages 10 to 80 to actively engage in climate-related endeavors and take pride in the badges they earn.

Anticipated outcomes include the establishment and growth of a community of climate achievers comprising staff, professors, researchers, and students. This community, acknowledged through the OpenPass4Climate platform without borders, is expected to expand beyond university boundaries to fulfill the aspirations of students seeking Open Badges for their ongoing contributions and to facilitate the acquisition of new knowledge while actively engaging in positive actions.

To effectively identify and evaluate diverse climate commitments, it is imperative to collect information concerning the nature of students’ engagement with climate-related activities. This process encompasses both implicit and explicit forms of recognition.

It is important to acknowledge, however, that recognition systems embody particular assumptions about human motivation that merit critical examination. Research in motivational psychology demonstrates that humans act based on their personal meanings and interpretations rather than simply responding to external stimuli ( Ryan & Deci, 2000). This raises important questions for digital credentialing in climate education. While badges and micro-credentials function as external motivators, extensive research shows that extrinsic rewards can undermine intrinsic motivation when perceived as controlling rather than informational ( Deci *et al.*, 1999). Not all learners will value such recognition equally—some may pursue badges regardless of genuine environmental commitment, while others deeply engaged in climate action may find them irrelevant to their values. Understanding these motivational dynamics is essential for designing recognition systems that support rather than undermine authentic environmental engagement.

### The rise of open recognition systems

The landscape of educational credentialing is undergoing significant transformation. While conventional academic credentials retain their significance, the educational landscape now embraces more nuanced and adaptable recognition mechanisms. These emerging approaches provide enhanced capacity to document and validate the multifaceted competencies developed across structured educational programs, community-based learning contexts, and self-directed knowledge acquisition pathways. Open badges and micro-credentials have emerged as prominent tools in this evolution, offering verification mechanisms for skills and knowledge that might otherwise go unrecognized ( West & Cheng, 2023; Zawacki-Richter *et al.*, 2020; Wolz *et al.*, 2021).

Open recognition systems refer to flexible, technology-enabled frameworks—such as open badges and micro-credentials—that validate learning across formal, non-formal, and informal contexts. These systems enable learners to receive recognition for discrete skills and achievements, regardless of where or how the learning occurred.

While the terms “open badges” and “digital badges” are often used interchangeably, it is important to note their distinctions. “Open badges” specifically refer to a standardized format for digital credentials, such as those adhering to the Mozilla Open Badges standard, which ensures interoperability and portability across platforms. “Digital badges,” on the other hand, is a broader term encompassing any digital representation of skills or achievements, which may or may not follow a specific standard. In the context of climate education, both open badges and broader digital badges offer promising solutions for recognizing interdisciplinary competencies and action-oriented achievements.

The concept of open recognition for learning achievements has gained significant traction. This development is particularly evident as educational pathways become more diverse. Additionally, the workforce increasingly demands more specific and demonstrable competencies. This trend has accelerated since the COVID-19 pandemic, which highlighted the need for flexible, accessible learning options and rapidly verifiable skills ( Tamoliune *et al.*, 2023). For climate education specifically, these systems offer the potential to recognize the multidisciplinary knowledge and action-oriented competencies that are essential for addressing complex environmental challenges.

### The relevance to climate education

Climate change education presents unique challenges that make it particularly suited to open recognition approaches. First, climate literacy spans multiple disciplines, from natural sciences to social studies, policy, economics, and beyond. Traditional educational structures often struggle to integrate this interdisciplinary knowledge effectively. Second, addressing climate change requires not just knowledge but action and civic engagement, which are rarely recognized in conventional academic credentials. Third, climate education happens across multiple contexts—formal classrooms, community initiatives, workplace projects, and personal advocacy—creating a need for recognition systems that can bridge these varied learning environments.

Open badges and micro-credentials offer promising solutions to these challenges. They can verify discrete competencies across disciplinary boundaries, recognize action-oriented achievements beyond knowledge acquisition, and validate learning regardless of where it occurs ( Cumberland *et al.*, 2023). This alignment between the needs of climate education and the affordances of open recognition systems suggests significant potential for their application in this domain.

### Purpose and scope of this review

This literature review aims to investigate current practices in open recognition systems, with a particular focus on their application and potential in climate education. It is conducted as part of Work Package 3 (WP3) of the OpenPass4Climate Erasmus+ project, which seeks to identify and evaluate different climate commitments and assess students’ perceptions of their needs through an open recognition framework.

Specifically, this review addresses the following objectives:

– To examine the main contributions in the field of open recognition learning, particularly focusing on badges, micro-credentials, and their potential application in climate education contexts;
– To identify key challenges in implementing open recognition systems for climate-related competencies and actions; and
– To synthesize recommendations from existing research for the effective implementation of open badges and micro-credentials in climate education.

By addressing these objectives, this review aims to provide a comprehensive foundation for the development of the OpenPass4Climate system, informing both the conceptual framework and practical implementation of open badges for recognizing climate action and learning.

## Method

### Research design

This study is a scoping review designed to map the existing literature on open recognition systems and their application to climate education. We chose this methodology, following the five-stage framework proposed by Arksey & O’Malley (2005) and further refined by Levac *et al*. (2010), as it is ideal for exploring emerging fields, clarifying key concepts, and identifying research gaps from a diverse body of evidence. Unlike a systematic review, which typically focuses on a narrow question and homogenous study types, a scoping review allows for the inclusion of varied literature, including the ‘grey literature’ essential for understanding this practice-driven field.

Guided by this scoping review design, we addressed the following research questions: (RQ1) How are open recognition systems (including digital badges, micro-credentials, and OER-related recognition) currently conceptualized and implemented across educational and professional contexts? (RQ2) To what extent are these systems being applied to climate education and eco-pedagogical activities, and what forms do such applications take? (RQ3) What challenges, tensions, and opportunities does the literature identify regarding the use of open recognition systems to support climate-related competencies and action?

### Search strategy and sources

***Databases***

To ensure comprehensive coverage of the literature, the following databases were searched:

– Web of Science
– EBSCOhost
– ERIC (Education Resources Information Center)
– Scopus
– Google Scholar
– Wiley
– PubMed/Europe PMC
– SemanticScholar

The selection of databases was deliberate and methodologically sound. Google Scholar was included for its comprehensive coverage across disciplines and ability to index a wide range of publication types, including those not captured by traditional academic databases. ERIC (Education Resources Information Center) was selected for its specialized focus on education literature, making it particularly valuable for identifying research on educational badges and credentials. Web of Science and Scopus were chosen for their rigorous indexing standards and citation tracking capabilities, facilitating the identification of highly cited, influential works in the field. The inclusion of EBSCOhost, Wiley, PubMed/Europe PMC, and SemanticScholar further ensured the breadth of coverage across different publishers and disciplinary perspectives, which is particularly important given the interdisciplinary nature of climate education and open recognition systems.

Additionally, we conducted searches in French, Portuguese, Italian, and German language sources to capture relevant literature from multiple linguistic contexts.

***Search terms and query development***

The search strategy was developed through an iterative process, focusing on the intersection of open recognition systems and climate or environmental education. The primary search strings used included:

• (“open education recognition system” OR “open badges” OR “digital badges” OR “micro-credentials”) AND (“climate change” OR “environmental actions” OR “ecopedagogical activities”)

Additional search strings were developed to refine results by educational context:

• (“open education recognition system” OR “open badges” OR “digital badges”) AND (“climate change” OR “environmental actions” OR “ecopedagogical activities”) AND (“K-12” OR “higher education” OR “lifelong learning” OR “general public”)

Further search strings focused on methodological aspects, best practices, and international perspectives:

• (“open education recognition system” OR “open badges” OR “digital badges”) AND (“climate change” OR “environmental actions” OR “ecopedagogical activities”) AND (methodology OR approach OR implementation)
• (“open education recognition system” OR “open badges” OR “digital badges”) AND (“climate change” OR “environmental actions” OR “ecopedagogical activities”) AND (“best practices” OR “successful cases” OR “exemplary models”)
• (“open education recognition system” OR “open badges” OR “digital badges”) AND (“climate change” OR “environmental actions” OR “ecopedagogical activities”) AND (“international perspective” OR “global initiatives” OR “cross-cultural”)

These search strings were adapted as needed for each database and translated for non-English language searches. All search strings and results were documented to ensure transparency.

### Search results

Initial database searches yielded a significant number of records across all databases. After applying filters for publication date (2010–2024), peer-reviewed status, and removing duplicates, 477 unique records were identified for initial screening.

### Selection criteria and process

***Inclusion criteria***

Studies were included if they met the following criteria:

– Published between January 1, 2010, and November 30, 2024
– Published in peer-reviewed journals, academic book chapters, institutional or governmental reports from recognized organizations, or peer-reviewed conference proceedings. This inclusive approach was adopted because much of the innovation in open recognition is driven by practice and documented in ‘grey literature’ that is crucial for a comprehensive understanding of implementation challenges and opportunities
– Available in full-text format (with university library membership)
– Focus on digital badges, open badges, or micro-credentials in educational or professional development contexts
– Keywords mentioned in the title or abstract

***Exclusion criteria***

Studies were excluded if they:

– Referred to digital badges when they were not considered as micro-credentials
– Lacked substantive discussion of open recognition systems or their application

***Screening and selection process***

The selection process consisted of an initial screening of titles and abstracts against the inclusion and exclusion criteria, followed by a full-text review of the remaining articles. This process resulted in a final sample of 70 articles, review papers, book chapters, and reports for analysis.

***Data analysis***

The analysis employed a thematic synthesis approach to identify key themes, challenges, and recommendations related to open recognition systems for climate education. Following data extraction into a structured matrix capturing publication type, educational context, recognition tool type, climate/environmental focus, and reported outcomes, we conducted an inductive coding process to identify recurring concepts related to implementation strategies, barriers, motivational factors, and pedagogical considerations. Codes were iteratively grouped into higher-order themes (e.g., “current practices”, “technological and institutional barriers”, “motivational and theoretical considerations”, “eco-pedagogical gaps”) that provide the organizing structure for the Findings, Challenges, and Recommendations sections. Theme boundaries were discussed among the author team until agreement was reached. This qualitative synthesis aligns with the descriptive-analytical approach recommended for scoping reviews ( Levac *et al*., 2010).

### Limitations

Several limitations should be acknowledged:

– The review covers the literature published until January 31, 2025. To maintain methodological transparency and reproducibility, this search window was established *a priori* and was not extended during subsequent manuscript revisions.
– Despite our multilingual approach, language barriers may have affected the comprehensiveness of non-English literature coverage.
– The keyword search requirements may have excluded relevant articles that addressed related concepts but used different terminology.

These limitations notwithstanding, this review provides a solid foundation for understanding the current state of research on open recognition systems for climate education within the context of the OpenPass4Climate project.

The distribution of our reviewed literature is summarized in Table 1. The predominance of general education sources (88.6%) compared to climate-specific publications (11.4%) reflects the current state of the field and reinforces our finding that the application of open recognition systems to climate education remains largely unexplored.

**Table 1.** Distribution of reviewed literature.

Source type	Number	Key outlets	Year range	Climate/environment focus
Journal Articles	56	British Journal of Educational Technology (5), Educational Technology Research & Development (3), Open Praxis (3), Australasian Journal of Educational Technology (2), TechTrends (2), Journal of Teaching and Learning for Graduate Employability (2)	2009–2024	5 articles ( Annelin & Boström, 2022; Jirgensons & Kapenieks, 2018; Miller & Jorre de St Jorre, 2024; Ramirez-Montoya, 2020; Ramirez-Montoya *et al.*, 2022)
Book Chapters	3	Springer (3)	2021–2024	1 ( Halttunen *et al.*, 2024)
Reports/White papers	3	Royal Roads University (2), Deakin University (1)	2019–2021	2 ( Cox, 2021; Cox *et al.*, 2020)
Conference Proceedings	7	IEEE (1), CEUR (1), AAACE (1), iConference (1), Others (3)	2013–2022	0
Preprint	1	Qeios	2024	0
Total	70		2009–2024	8 (11.4%)

## Current practices in open recognition systems

As shown in Table 1, our analysis of 70 publications revealed extensive development in open recognition systems generally, but limited application to climate education specifically. Only eight publications (11.4%) directly addressed environmental or climate contexts, while the remainder discussed general educational applications. This disparity itself constitutes an important finding, suggesting that despite the urgent need for climate education and the affordances of digital recognition systems, their intersection remains largely unexplored. We present both the general developments and the limited climate-specific applications below, as understanding the broader landscape is essential for identifying opportunities for climate education integration.

### Badges and micro-credentials

Digital badges and micro-credentials are increasingly vital for recognizing and validating skills across formal and informal learning contexts, particularly in climate education.

It is important to clearly distinguish between micro-credentials and open badges, as these terms are sometimes used interchangeably but represent distinct concepts. Micro-credentials are broader certification units that validate specific skills, knowledge, or competencies, typically requiring assessment and often carrying academic credit value. They represent smaller learning achievements than full degrees and may be stackable toward larger qualifications. Open badges, in contrast, are a specific technological implementation—a digital representation of skills, achievements, or qualities that contains verifiable metadata about the earner, issuer, criteria, and evidence. While all open badges could be considered micro-credentials, not all micro-credentials are implemented as open badges. The key distinction lies in their scope and technical implementation: micro-credentials focus on the certification framework and academic recognition, while open badges emphasize the digital, portable, and verifiable representation of achievements across platforms.

Oliver (2019) defines micro-credentials as certifications of assessed learning that complement or extend formal qualifications. West and Cheng (2023) emphasize their role in open education infrastructure, highlighting affordances such as granular recognition of accomplishments, mastery demonstration, and lifelong portfolio creation, which are especially relevant for documenting climate-related competencies. Similarly, Wolz *et al.* (2021) underscore their potential to digitize traditional graduation certificates, aligning with employers’ digital recruitment processes and enhancing trust through secure, verifiable formats—attributes that could certify climate education achievements effectively.

A systematic review by Cumberland *et al.* (2023) found a growing interest in micro-credentials, particularly in higher education and workplace settings, where they can function either as pedagogical tools or as credentials that validate competencies. Digital badges represent a specific form of micro-credentialing that is gaining attention among employers and educational institutions alike.

Micro-credentials are seen as a means of certifying smaller attainments of learning than that of a full degree. This insight includes stackable credit, general recognition of prior learning, evidence of graduate attributes, and standards-based competencies associated with professional practice, promoting a structured approach to skills validation that bridges the gap between academic learning and professional demands ( Harris & Wihak, 2018). Authors assessing implementation and perceptions, such as Jirgensons and Kapenieks (2018) and Korhonen *et al.* (2020), found open badges useful for demonstrating competencies in job applications. However, Perkins and Pryor (2021) examined employer awareness and acceptance of digital badges, finding a significant gap in understanding that needs to be addressed for broader adoption. Dyjur and Lindstrom (2017) examined perceptions of digital badges, finding both positive and negative views. Davis and Singh (2015) identified badges as an opportunity to establish learner credibility outside of the original context but noted credibility as a dominant challenge requiring recognition by external audiences.

Micro-credentials demonstrate significant utility in domains outside traditional educational settings. Research conducted by Selvaratnam and Sankey (2021) identifies these compact certifications as offering Australian higher education institutions viable strategic directions for long-term sustainability, with particular effectiveness demonstrated in abbreviated learning modules and advanced graduate-level educational offerings. Similarly, a review by Varadarajan *et al.* (2023) suggests that micro-credentials can function as learning pathways, employment pathways, and qualification frameworks, serving the needs of different stakeholders, including learners, educational institutions, employers, and governmental bodies.

According to Mah (2016), integrating micro-credentials into institutional programs can enhance their value and utility, making them more accessible and impactful for all stakeholders. Pollard and Vincent (2022) also proposed to align principles of micro-credentials with university missions and embed them into the curriculum. However, in this higher education context, Trepulé *et al.* (2021) emphasized the importance of clear evaluation and recognition criteria for digital badges to increase their value for assessment and recognition. In this line, the framework for the design and development of open badge systems proposed by Devedžić and Jovanović (2015) can be particularly helpful.

A recent perspective piece highlights how digital badges can offer self-guided learning options that allow individuals to develop both professionally and personally at their own pace. Currie *et al.* (2023) highlight how digital credentialing systems function as intrinsic motivation catalysts, stimulating investment in ongoing professional growth. These systems additionally create beneficial competitive dynamics that enhance learning outcomes, particularly when participation remains voluntary—a condition that promotes increased innovative thinking, deeper interaction with educational content, and heightened learner fulfillment.

These positive findings must be interpreted alongside research showing more complex motivational dynamics. Meta-analyses of gamification in education, which includes badge systems, reveal highly variable effects on motivation and engagement ( Dichev & Dicheva, 2017). The impact appears to depend on whether badges are perceived as supporting competence development (informational) or as controlling behavior through rewards. When badges reduce complex learning to simple task completion, they may undermine the development of deeper understanding and commitment. This distinction is particularly relevant for climate education, where the goal extends beyond behavioral compliance to fostering enduring environmental values and identity.

Empirical research demonstrates the substantial motivational influence of open badge systems within recognition-based learning environments ( Abramovich *et al.*, 2013). Investigations by Cheng *et al.* (2018) reveal how digital credentialing mechanisms strengthen learners’ self-regulation capabilities by supporting structured objective formulation, enhancing commitment to established targets, managing assignment difficulty levels, and integrating evaluative input. Research further suggests that shifting from traditional grading approaches to open badge frameworks redirects learner attention toward comprehensive skill acquisition and knowledge development ( Tomić *et al.*, 2024). Carey and Stefaniak (2018) found that skill-based badges are more valuable than participation badges.

In examining specific applications relevant to climate education, three studies offer insights, though only Miller and Jorre de St Jorre (2024) directly address environmental contexts. West and Cheng (2023) present a comprehensive framework for how open microcredentials support personalized learning across micro-, meso-, and macro-levels. At the micro-level, they identify four mechanisms through which badges support self-regulated learning: (1) helping learners set explicit goals through clear badge criteria, (2) establishing prerequisite pathways that scaffold complex learning, (3) connecting learning goals to professional development outcomes, and (4) providing both summative and formative feedback through badge metadata. They emphasize that microcredentials can “break content into small modular units, so learners can create their own learning pathways”, with learners able to “hop on and off ‘the formal education bus’ when needed”. While not specific to climate education, their framework suggests how environmental learning could be modularized into stackable credentials addressing different competencies. For example, a learner could earn badges for ‘Carbon Footprint Analysis,’ ‘Stakeholder Communication,’ and ‘Policy Advocacy,’ creating a personalized pathway towards a ‘Climate Action Leadership’ micro-credential that is both recognizable and meaningful.

The most directly relevant empirical study is from Miller and Jorre de St Jorre (2024), who examined employer perspectives on digital micro-credentials in environmental science through interviews with 22 environmental professionals in Australia. Their work provides clear evidence of strong employer demand for verifiable skills highly relevant to climate action, including:

– Communication and collaboration: the ability to “work collaboratively… [and] maintain a sense of where that sits in terms of the overall effort”.
– Technical field skills: including environmental impact assessment, species identification, and data collection
– Systems thinking: the ability to understand “interconnections between social, economic, and environmental systems”
– Adaptability and lifelong learning: Employers emphasized the importance of “the want to keep learning and the ability to keep learning”.

Crucially, while employers were enthusiastic, they demanded rigor and clarity, with one stating, “unless we’re educated around what that symbol was actually around and the level of effort and rigour… then it doesn’t have the obvious impact.” This underscores the critical need for industry partnerships and transparent assessment standards for any climate-related credential.

Finally, Rollin (2021), though not focused on climate, offers a practical model for assessing the ‘transversal skills’ that are central to effective climate action. The study examines the ‘Garantie Jeunes’ program in France, which uses non-formal learning contexts to develop competencies like teamwork, autonomy, and responsibility in young adults (a full list of skills assessed is provided in the paper’s appendix, Table A1). While the paper does not offer detailed assessment rubrics, it highlights a methodology for recognizing skills developed through practical experience and structured reflection. This approach serves as a direct model for recognizing skills gained through community-based climate projects, advocacy, or volunteerism—activities that are rich in learning but often remain un-credentialed.

Recent applications in Nordic coastal tourism education further demonstrate the practical value of micro-credentials and open badges in sustainability contexts. Halttunen *et al.* (2024) highlight how these credentials provide flexible, cost-effective pathways for tourism workers to develop sustainability competencies despite geographical constraints in remote destinations. Their research found that both tourism companies and destination management organizations recognized the complementary value of industry certificates and open badges for verifying skills in sustainability practices, with certificates meeting operational standards and badges demonstrating advanced expertise development. This application suggests that open badges could bridge formal education with practical environmental outcomes, enhancing learner engagement across diverse contexts, including the climate education field.

Additionally, Cox *et al.* (2020), cited in the ALN Climate Adaptation Micro-credential Strategy ( Cox, 2021), developed an openly licensed Climate Adaptation Competency Framework that aligns with micro-credential programs targeting professionals who need climate adaptation capabilities. Their framework identifies five competency domains with five sub-competencies each, structured to support criterion-based professional development in climate adaptation. This approach demonstrates how micro-credentials can be strategically designed to address workforce gaps in climate knowledge and skills, particularly in areas requiring strategic foresight for adaptation challenges.

### Open Educational Resources (OER) and Practices (OEP), MOOCs, and online learning

The adoption of open educational resources (OER) and open educational practices (OEP) has been widely explored, offering substantial benefits for enhancing learning accessibility and quality, particularly in climate education. West and Cheng (2023) argue that OER, when paired with open micro-credentials, forms a comprehensive open education infrastructure that supports personalized learning pathways—an approach that could democratize access to climate literacy.

Littlejohn and Hood (2017) demonstrated that OER engagement promotes three levels of learning for educators, which can be leveraged to structure workplace learning opportunities. van de Laar *et al.* (2024) found that open-access online courses were valued by Ph.D. students, although open micro-credentials did not incentivize course completion for this population. Bozkurt *et al.* (2019) and Knox (2013) highlighted the growing importance of openness in education, while also noticing challenges such as the divide between the global north and south in research output and the need for a critical exploration of the relationships between technology and education. Jung and Lee (2020) examined cultural factors influencing OER adoption and found that habit is a strong determinant across cultures, although other factors such as performance expectations and social influence also play a critical role. Bossu and Fountain (2015) introduced an open micro-course to build capacity in OEP. Adedoyin and Altinay (2023) examined factors that influence students’ intentions to use OER. Knox (2013) proposed the inclusion of “open processes” in OEP. Ramirez-Montoya (2020) discussed how open education can support educational processes. Open science and scholarship promote transparency, accessibility, and collaboration in research. Corrall and Pinfield (2014) discussed the theoretical underpinnings of openness in different domains, emphasizing the importance of an integrated approach to open practices. Littlejohn and Hood (2017) studied how educators learn through engagement with OER, demonstrating the transformative potential of open educational resources in professional development. Askeroth and Newby (2020) noted that badges are well-suited for adult learners in less formal educational settings.

Massive open online courses (MOOCs) have been instrumental in providing scalable and flexible learning opportunities. The most significant contribution came from Ramirez-Montoya *et al.* (2022), who identified key factors influencing MOOC effectiveness, such as perceived usefulness, self-efficacy, and achievement motivation, which are critical to designing effective online learning experiences. Gates and Walters (2015) explored the relevance of MOOCs in social work education, highlighting their potential in delivering professional training. Kopp and Ebner (2017) found that the awarding of certificates has an impact on MOOC learners, but the impact varies depending on the target group, commitment, and usability. Other studies have contributed by examining approaches to assessment, such as Farrow *et al.* (2021), who proposed identity verification strategies for MOOC platforms, and Chauhan (2014), who discussed automated assessment techniques in MOOCs. “Smart systems” are being developed to track and predict learner behavior. Bossu and Fountain (2015) acknowledge that Applaud —a specific online scheme system to gain recognition— has had a positive impact on learning and teaching at the Open University, supporting professional development, career development, and reflective practice. Charleer *et al.* (2013) found that gamified dashboards such as Navi Badgeboard helped with awareness of personal activities, while Navi Surface improved collaboration and reflection.

Further applications of OER and MOOCs in climate education underscore their synergy with open recognition systems. Cieply and Grand (2019) analyzed the use of open badges in higher education to recognize competencies developed through OER-based learning, offering a scalable model for climate education that fosters critical engagement with ecological issues. This complements the emphasis of West and Cheng (2023) on micro-credentials supporting inquiry-based learning, which could enhance climate-focused curricula across educational levels.

Yet the promise of democratization through OER and open recognition requires critical examination. Access to digital technologies and stable internet remains unequally distributed globally, potentially excluding the very communities most affected by climate change from these educational opportunities. Moreover, the emphasis on self-directed online learning assumes learners have the time, resources, and cultural capital to navigate these systems independently. Without addressing these structural inequalities, open recognition systems risk creating new forms of exclusion even as they promise greater accessibility.

### Recognition of prior and non-formal learning

Regarding the recognition of prior and non-formal learning, Schmidt *et al.* (2009) proposed a peer-based method of assessment and recognition that uses online communities to provide a credible alternative to traditional assessment methods. Tereseviciene *et al.* (2020) studied the recognition of open online learning in higher education and identified challenges in adapting to changes in the labor market. Friesen and Wihak (2013) explored the links between open educational contexts and prior learning assessment and recognition (PLAR). Harris and Wihak (2018) identified synergies in the recognition of non-formal learning. The integration of technology in higher education is essential to adapt to the changing demands of the workforce. Therefore, Lang (2023) argued that universities should adopt digital micro-credentials to meet the changing needs of the workforce and facilitate a shift toward more flexible and relevant educational offerings. Orr *et al.* (2019) identified archetypes of technology-enabled higher education, providing a framework for understanding different levels of digital adoption in institutions. Volungevičienė *et al.* (2020) supported the shift to learner-centered, multidimensional teaching models that leverage new digital devices to create more engaging and effective learning environments. Zawacki-Richter *et al.* (2020) provided a comprehensive review of open and distance learning research, identifying trends and gaps in the current landscape.

The COVID-19 pandemic has accelerated the adoption of distance education across all types of schools, creating both opportunities and challenges for Open Universities ( Lyu, 2023). While this expansion has made micro-credentials more relevant, it also raises concerns about how to effectively implement online teaching and maintain educational quality. The pandemic has stimulated the need for developing new competencies and personalized, competency-based, flexible, and collaborative professional development ( Varadarajan *et al.*, 2023). These changes have highlighted the importance of micro-credentials as tools for rapid upskilling in an educational landscape that increasingly embraces online delivery modalities.

Another contribution of reviewed research on open learning is the recognition of competency frameworks and sustainability in education, which are critical to aligning skills with evolving industry needs and promoting lifelong learning. Fitsilis (2024) emphasized the importance of competency frameworks in Education 4.0 that align individual skills with organizational goals. Annelin and Boström (2022) analyzed questionnaire assessment tools for measuring sustainability competencies and identified the need for comprehensive assessment tools that span multiple disciplines.

However, these frameworks for recognizing non-formal learning rarely examine fundamental questions about what knowledge is deemed worthy of recognition and by whom. The selection of competencies to recognize inevitably involves value judgments about what constitutes legitimate knowledge. In climate education, this raises important questions: How do recognition systems account for indigenous knowledge systems that have sustained environmental balance for generations? Can they capture the embodied knowledge of communities experiencing climate impacts firsthand? The absence of these discussions in the reviewed literature suggests that despite rhetoric of openness and inclusivity, recognition systems may primarily validate knowledge that fits within established academic and professional frameworks.

### The eco-pedagogy gap: A critical absence in digital recognition

Our search for literature combining ‘eco-pedagogical activities’ with open recognition systems yielded zero results, despite eco-pedagogy’s growing importance in climate education. This absence is particularly striking given the theoretical alignment between eco-pedagogical principles and the affordances of digital credentials.

Eco-pedagogy, rooted in Paulo Freire’s critical pedagogy ( Freire, 1970), aims to develop “planetary consciousness” and foster social and environmental justice through transformative educational practices ( Gadotti, 2008; Kahn, 2010). Its methods include experiential learning, place-based education, systems thinking workshops, and action-oriented climate projects ( Hung, 2021; Kopnina, 2020; Misiaszek, 2020). These activities emphasize critical consciousness, praxis (reflection and action), and transformative learning—elements that could be recognized through carefully designed digital credentials.

The complete absence of eco-pedagogical frameworks in the digital recognition literature suggests several profound barriers: a philosophical tension between the standardization required for badges and the contextual, transformative nature of eco-pedagogy ( Andreotti, 2006); the inherent difficulty in assessing outcomes like ‘critical consciousness’ and ‘planetary citizenship’ ( Misiaszek, 2018); the privileging of technical competencies over the critical, values-based learning central to eco-pedagogy in current badging systems ( Kopnina, 2020); and a likely disconnect between the educational technology and critical pedagogy communities.

This gap has significant implications. While institutions implement diverse eco-pedagogical activities—from climate action workshops to community-based environmental projects—these transformative learning experiences remain unrecognized in formal credentialing systems. This invisibility may perpetuate the dominance of technical approaches to climate education over critical, action-oriented pedagogies.

The absence of eco-pedagogical approaches in digital recognition systems represents more than a technical gap—it reflects deeper tensions in how we conceptualize and value environmental learning. This tension reflects fundamental incompatibilities between the standardizable nature of digital credentials and the context-dependent, transformative goals of eco-pedagogy. As Misiaszek (2018) argues, eco-pedagogy is “essentially literacy education for reading and re-reading human acts of environmental violence.” Such critical analysis resists easy codification into badge criteria. Eco-pedagogical approaches emphasize critical consciousness and praxis—the integration of reflection and action ( Freire, 1970). How does one assess ‘planetary consciousness’ or ‘environmental justice awareness’ through standardized metrics? The challenge is not merely technical but epistemological, requiring recognition systems that can value process and transformation alongside measurable outcomes.

More fundamentally, this incompatibility raises questions about whether digital credentials might actually work against eco-pedagogical goals. If eco-pedagogy aims to develop critical consciousness about power structures perpetuating environmental destruction, how can this be reconciled with credentialing systems that potentially reproduce those same power structures? The very act of reducing complex environmental consciousness to a series of badges might represent what Freire called ‘banking education’—depositing information rather than fostering critical dialogue. This suggests that rather than trying to force eco-pedagogy into credentialing frameworks, we might need to question whether some forms of transformative learning should remain outside systems of formal recognition. The absence of eco-pedagogical approaches in digital credentialing literature might not be a gap to be filled, but rather a reflection of fundamental incompatibility that should give us pause. Yet this challenge also presents an opportunity.

Digital recognition systems could evolve beyond competency verification to recognize transformative learning processes through badges that document critical reflection on environmental justice issues, credentials recognizing sustained community environmental action, portfolios capturing the development of “planetary citizenship” ( Gadotti, 2008), and recognition for facilitating others’ environmental conscientization. The OpenPass4Climate project is uniquely positioned to bridge this gap by developing recognition frameworks that honor both the measurable and transformative aspects of climate education.

## Motivational and theoretical considerations

### The complexity of human motivation in recognition systems

The reviewed literature reveals limited engagement with fundamental questions about how recognition systems interact with human motivation. A fundamental paradox emerges: if genuine climate action requires intrinsic motivation and deep personal transformation, why implement external recognition systems at all? This question deserves direct acknowledgment. The honest answer is that we face a tension between the ideal of purely intrinsic motivation and the practical reality that educational and professional systems currently operate through credentials. Rather than resolving this tension, we must work within it, remaining vigilant about the risks while exploring whether recognition systems can be designed to support rather than undermine authentic engagement. Self-Determination Theory (SDT) provides a useful framework for understanding these dynamics ( Ryan & Deci, 2000). SDT distinguishes between different types of motivation along a continuum from external regulation (behavior driven by external rewards or punishments) to integrated regulation (behavior aligned with one’s values and identity). This distinction is crucial for climate education, where the goal is to develop internalized commitment rather than temporary compliance.

Research demonstrates that external rewards can undermine intrinsic motivation through a process called the ‘overjustification effect,’ particularly when rewards are perceived as controlling ( Lepper *et al.*, 1973). In educational contexts, this can lead learners to focus on obtaining credentials rather than developing genuine understanding. For climate education, this presents a significant challenge: badges intended to motivate environmental action might inadvertently reduce complex environmental engagement to a series of trackable tasks, potentially alienating learners whose commitment stems from intrinsic values.

However, SDT also suggests that external recognition can support motivation when it provides informational feedback about competence development rather than controlling evaluation ( Deci & Ryan, 1985). Abramovich *et al.*, 2013 found that badges’ impact depends on both badge type and learner expertise, with skill-based badges providing more value than participation badges. This suggests that carefully designed recognition systems might support rather than undermine motivation, but only if they align with learners’ psychological needs for autonomy, competence, and relatedness.

***Deep versus shallow engagement in climate education***

The distinction between deep and shallow cognitive engagement is particularly critical in climate education. Shallow engagement manifests as learners completing required activities to earn badges without internalizing environmental values or understanding systemic issues. For example, a student might complete a ‘Energy Saver’ badge by documenting reduced electricity use for a week, then immediately revert to previous consumption patterns. This represents what Kohn (1999) calls “compliance without commitment”—the learner performs the visible behavior without developing understanding of energy systems, climate impacts, or personal responsibility.

Deep engagement, by contrast, involves learners questioning their assumptions, understanding interconnections between personal actions and global systems, and developing lasting commitment to environmental justice. This might manifest as a learner who, while pursuing energy-related learning, begins investigating their community’s energy infrastructure, questioning why certain neighborhoods lack access to renewable options, and organizing collective action for change. Such engagement cannot be easily captured in badge criteria because it involves emergent, unpredictable learning paths driven by genuine curiosity and concern.

The risk is that badge systems, by defining specific criteria for recognition, may inadvertently encourage shallow engagement by making the credential rather than the learning the goal. This represents a behavioristic approach—focusing on observable behaviors rather than internal transformation.

### Meaning-making and individual differences

People act based on their meanings and interpretations, which highlights another limitation in current recognition systems. Constructivist learning theory emphasizes that knowledge is actively constructed through interaction with one’s environment and social context ( Vygotsky, 1978). In climate education, this means the same badge may hold vastly different meanings for different learners based on their cultural background, prior experiences, and value systems.

This variability in meaning-making is particularly relevant for environmental behavior. Kollmuss and Agyeman (2002) identify numerous factors beyond external incentives that influence pro-environmental behavior, including emotional involvement, locus of control, and cultural values. A badge recognizing ‘Carbon Footprint Reduction’ might be meaningful to someone already committed to individual environmental action but could feel irrelevant to someone whose cultural context emphasizes collective responsibility for environmental issues.

### Implications for climate education recognition

These theoretical considerations suggest that recognition systems for climate education face inherent tensions. On one hand, standardized credentials offer potential benefits for validating learning and facilitating mobility across educational contexts. On the other hand, the standardization required for portability may conflict with the personalized, value-based nature of environmental commitment. The challenge is not whether to use digital credentials, but how to design them in ways that:

Provide informational feedback about developing competencies without becoming controlling mechanisms

– Allow for diverse interpretations and pathways that reflect different cultural contexts and values
– Support community and collective achievement rather than only individual accomplishment
– Connect to real-world impact and meaningful action rather than abstract task completion

The limited attention to these considerations in the reviewed literature suggests that current implementations may not fully account for the complexity of human motivation in environmental contexts.

The tendency to accept digital credentials as inherently beneficial, evident in much of the reviewed literature, overlooks these complexities. A more critical approach reveals that recognition systems embody particular values and assumptions about learning, achievement, and credibility. They privilege certain forms of knowledge over others, potentially marginalizing alternative ways of knowing and being that are crucial for addressing complex environmental challenges. Future research and implementation must move beyond technical questions of how to implement badges to fundamental questions about what purposes they serve and whose interests they advance.

## Challenges

### Standardization and definitional issues

The lack of standardized definitions and criteria for openness leads to inconsistent results. Different interpretations of what constitutes open learning recognition can lead to differences in implementation and effectiveness, as noted in several studies.

A persistent challenge in the micro-credential landscape is the absence of global standardization, complicating their application in climate education. For instance, a climate adaptation course might be valued differently across regions due to varying credit definitions, hindering learners’ ability to transfer credentials internationally. Ahsan *et al.* (2023) note the lack of consensus on micro-credential definitions and credit values across universities, industries, and governments, a concern echoed by Wolz *et al.* (2021), who identify terminological ambiguity in higher education institutions as a barrier to adoption. For example, the European Credit Transfer System defines micro-credentials as conferring a minimum of 5 ECTS credits, while the New Zealand Qualifications Authority bounds them between 5 and 40 credits ( Varadarajan *et al.*, 2023).

### Institutional coherence and coordination

One of the challenges reported in the reviewed studies was the lack of coherence and coordination between different open initiatives. The need for a unified policy approach to integrate different open domains and maximize their potential benefits for learners has been highlighted as a critical concern ( Corrall & Pinfield, 2014). It has also been mentioned as a limitation in the widespread adoption and implementation of open learning recognition tools that it requires significant cultural change within educational institutions.

### Stakeholder awareness and acceptance

Perkins and Pryor (2021) found a significant gap in employer awareness and acceptance of digital badges, indicating the need for broader educational efforts to increase understanding and acceptance in formal and informal learning environments. Miller and Jorre de St Jorre’s (2024) findings reinforce these challenges in the environmental sector specifically. They found that environmental employers often relied on proxy indicators like work experience and internships because they lacked familiarity with digital credentials. One employer admitted: “trying to get a feel for the person and their skillsets beyond the CV is part of the challenge”. This suggests that even in fields directly relevant to climate action, significant education and trust-building are needed before micro-credentials can fulfill their potential.

The research by Halttunen *et al.* (2024) additionally revealed unique challenges in implementing open badges for sustainability education in tourism, particularly in remote or seasonal destinations where access to training is limited by geographical and economic constraints. Their findings suggest that while digital credentials can overcome some of these barriers, they must be designed with awareness of contextual limitations like connectivity issues or variable workforce composition throughout the year.

Cumberland *et al.* (2023) conducted a systematic review of the digital badge landscape in educational and workplace settings, identifying two main uses: as pedagogical tools and as micro-credentials. Their work identifies persistent confusion in nomenclature and a divergence in findings that suggests the need for further exploration. The authors note that for digital badges to be valuable, employers must recognize them as such, which presents both a challenge and an opportunity for collaboration between educational institutions and the labor market.

### Technological and implementation barriers

In addition, technological challenges such as scalability, privacy, and storage capacity, particularly with blockchain technology, pose significant barriers. Jirgensons and Kapenieks (2018) discuss these issues in the context of implementing blockchain for digital learning. Korhonen *et al.* (2019) reported that learners attached earned open badges to their ePortfolios and shared them in digital environments to showcase their skills. Organizational barriers, including resistance from traditional academic and publishing sectors, also hinder the adoption of open practices. In addition to technological challenges, sharing research data and educational resources often requires additional effort, which can be a restraint for educators and researchers. The additional workload and lack of support mechanisms are significant barriers to wider adoption.

Research by Ahsan *et al.* (2023) also identified technological readiness as a critical factor in micro-credential implementation. Without supportive learning technology, implementing micro-credentials in higher education becomes impractical. The authors noted that blockchain technology could potentially enhance the authenticity of micro-credentials through certified digital documents, though further research on its adoption is needed. Wolz *et al.* (2021) discuss blockchain’s potential to enhance credential security, yet note scalability and privacy challenges that could hinder its use in certifying environmental achievements. A French report by Rollin (2022) identifies methodological issues—reliability, validity, and equity—in assessing transversal skills via open badges, underscoring the need for robust frameworks to ensure equitable recognition of climate education outcomes across diverse learners.

### Climate education-specific challenges

The Climate Adaptation Micro-credential Strategy ( Cox, 2021) also points to the challenge of creating comprehensive recognition systems that can effectively connect disparate educational offerings related to climate adaptation. The strategy highlights the need for constant interaction between didactics and professional practice to ensure that micro-credentials meet real-world needs in addressing climate challenges. This connects to broader concerns about fragmentation in sustainability education, where many initiatives exist but often lack coherent frameworks that allow for progressive skill-building across different contexts.

## Recommendations

The literature review highlights several key recommendations for the effective implementation of micro-credentials and digital badges. These include addressing the complex motivational dynamics between external recognition and intrinsic engagement; integrating them into institutional programs to ensure they add value and are easy to use with clear metrics; embedding them in the curriculum using critical and reflective pedagogy ( Mah, 2016; Pollard & Vincent, 2022); and making digital credentials easy to understand and valuable to employers and also aligning pedagogical practices with employer needs ( Perkins & Pryor, 2021). McNamara and Sun (2021) called for a global agreement on standards for the recognition, creation, validation, and distribution of micro-credentials and digital badges. However, as this review reveals, technical and institutional considerations must be balanced with careful attention to how these systems interact with learners’ intrinsic motivation and diverse cultural contexts.

### Addressing motivational complexity

Based on the theoretical considerations identified in this review, we recommend that recognition systems for climate education explicitly address motivational dynamics:

– Design for informational rather than controlling feedback: credentials should provide meaningful information about competency development rather than functioning as rewards for compliance. This involves detailed feedback mechanisms and clear connections between recognized skills and real-world environmental challenges.
– Allow for diverse pathways and interpretations: recognition systems should accommodate different cultural contexts and value systems rather than imposing uniform criteria. This might involve flexible frameworks that recognize various forms of environmental engagement.
– Emphasize collective alongside individual achievement: given climate change’s collective nature, recognition systems should validate collaborative action and community engagement, not only individual accomplishments.
– Connect recognition to meaningful action: badges should link to tangible environmental impact and ongoing engagement rather than one-time task completion, helping maintain the connection between recognition and genuine environmental commitment.

### Institutional integration and policy

The systematic analysis of digital credentialing practices by Cumberland *et al.* (2023) recommends that tertiary education organizations develop governance frameworks and institutional regulations prior to implementation. Their findings emphasize the necessity of creating structured classification systems that delineate how micro-credentials integrate within institution-specific educational frameworks. Their research identifies concentrated learning experiences and advanced degree programming as optimal starting points for micro-credential integration due to their reduced implementation risks. The authors highlight the importance of standardization to ensure portability between institutions, an aspect that has also been pointed out by other recent research, including McNamara and Sun (2021).

### Employer and stakeholder alignment

Recent reviews indicate that employers value micro-credentials as a way to fulfill their specific requirements and acknowledge skills using digital badges ( Varadarajan *et al.*, 2023). However, employers express concerns about the consistency of micro-credentials, their integrity, and the potential for fraudulent credentials. As noted by Kahle-Piasecki *et al.* (2024), the success of micro-credentials in the industry depends on standardization and recognition, suggesting the need for partnerships between higher education institutions, industry, and government agencies. This aligns with Perkins and Pryor (2021), who stress making credentials valuable and understandable to employers.

Building on Miller and Jorre de St Jorre’s (2024) environmental sector findings, partnerships should:

– Include employers in credential design to ensure relevance to workplace needs
– Provide clear metadata about assessment methods and standards
– Balance comprehensive evidence with reviewability constraints
– Emphasize industry endorsement to build trust
– Create mechanisms for employers to quickly understand credential value without an extensive portfolio review

### Technical and design aspects

*Badge system design.* Develop badge systems that provide visible recognition with metadata linked to validating evidence of educational achievement ( Gibson *et al.*, 2015; Gibson *et al.*, 2023). Design badge systems that encourage teamwork and public display of achievement ( Goodyear & Nathan-Roberts, 2017).

*Technical ecosystem.* Sousa-Vieira *et al.* (2021) suggest that the successful implementation of digital badges in higher education requires a comprehensive technical ecosystem. This includes considerations for storage, security, and administrative management of badges to maintain their integrity and authenticity. According to their conceptual framework, the badge module should include properties such as name, description, icon, text, visibility settings, and type (automatic, manual, or by contest).

*Blockchain solutions.* Wolz *et al.* (2021) propose blockchain-based systems to secure digital credentials, offering a scalable solution for documenting lifelong climate learning—a strategy that could build trust among EU stakeholders.

### Pedagogical approaches

Werth and Williams (2022) advocate building on open pedagogy to democratize learning experiences. McDaniel (2016) suggested the use of a design matrix and design recommendations for badges derived from diverse perspectives. Addressing cultural and contextual differences is essential to effectively adopt open learning recognition tools. Additionally, Mah (2016) and Pollard and Vincent (2022) emphasize embedding micro-credentials in the curriculum using critical and reflective pedagogy to enhance educational value.

***Ecopedagogical integration***

To bridge the identified gap, badge design for climate education should be guided by eco-pedagogical principles:

– Develop criteria that recognize praxis—the integration of critical reflection and environmental action ( Freire, 1970)
– Create pathways validating community-based environmental engagement and collective action, not just individual achievement.
– Design e-portfolios that capture transformative learning journeys, not just static outcomes
– Include peer recognition mechanisms reflecting eco-pedagogy’s emphasis on horizontal learning relationships.
– Integrate place-based learning achievements that connect global climate challenges to local contexts ( Sobel, 2004).

### Applications in climate education

*Environmental science context.* Based on their research in environmental science fields, Miller and Jorre de St Jorre (2024) recommend that micro-credential developers provide clear context about how these credentials can be used in professional settings and ensure rigorous assessment standards to build employer confidence. Their findings suggest that environmental employers value multiple lines of evidence when recruiting, and micro-credentials could serve as valuable supplementary validation of skills if properly contextualized.

*Nordic tourism education.* In the context of Nordic tourism education, Halttunen *et al.* (2024) suggest a blended learning approach that combines on-site workplace training with digital badges and MOOCs to accommodate learning in remote destinations. Their research emphasizes the importance of establishing common proficiency goals as frames of reference for sustainability competencies and providing flexible recognition mechanisms that can validate both existing and newly developed skills. This approach could be particularly relevant for climate education in contexts where geographical barriers limit access to traditional educational opportunities.

*Climate adaptation strategy.* The Climate Adaptation Micro-credential Strategy ( Cox, 2021) proposes a strategic approach for climate education that involves basing micro-credentials on open competency frameworks that clearly articulate required skills across different domains of climate adaptation work. Their recommendations include:

– Certifying courses using established competency frameworks to provide clear standards for employers and learners
– Designing micro-credentials for hybrid delivery with both synchronous and asynchronous components to accommodate working professionals
– Awarding credentials digitally with verifiable critical information summaries that provide metadata about the competencies achieved
– Creating pathways for prior learning assessment that allow experienced professionals to demonstrate existing climate adaptation skills

These recommendations are particularly relevant for developing open recognition systems in climate education as they address both pedagogical design and practical implementation concerns.

*Accessibility and learner agency.* To advance open recognition in climate education, both accessibility and learner agency should be prioritized. Jean-Marie and Millet (2022) advocate making badge tools accessible to all educational actors, ensuring equitable participation in climate-focused programs.

### Future research directions

Building on the findings and recommendations presented in the preceding sections, further research and development are essential to advance the recognition of open learning, particularly at its intersection with climate education. For instance, Jirgensons and Kapenieks (2018) suggest conducting case studies to explore the potential of blockchain technology in different courses and programs. Jung and Lee (2020) suggested conducting qualitative or mixed methods studies to understand educators’ use of OER within and across cultures. Lee (2020) recommended focusing efforts on opening higher education to disadvantaged groups and collecting real-life stories to inform practice. Randall *et al.* (2019) recommended further research to support the use of instructional design assistants for badge creation, as innovative pedagogical approaches and thoughtful design are key to successful implementation. The scholarly discourse surrounding recognition systems continues to evolve, with Roy and Clark (2019) particularly emphasizing the necessity for expanded investigation across varied educational and cultural environments. Contemporary research affirms the enduring relevance of digital credentialing mechanisms within educational innovation ( Farmer & West, 2016), yet significant knowledge gaps persist that require targeted investigation to advance understanding of their full potential and limitations.

## Conclusion

This review aimed to explore open recognition systems’ application in climate education but revealed a significant gap in this intersection. While open recognition systems show considerable promise in general educational contexts—with 70 publications documenting various implementations—only 8 (11.4%) specifically addressed environmental or climate contexts. This finding itself provides valuable insight into the current state of the field.

The limited examples we identified come primarily from Nordic tourism education and climate adaptation professional development. These suggest potential pathways through competency-based frameworks, blended learning approaches, and industry partnerships, but do not yet constitute proven models for widespread implementation. Most current applications focus on technical skills and knowledge verification rather than the transformative learning central to effective climate education.

Furthermore, our analysis reveals that most existing literature on recognition systems does not adequately address the complex relationship between external credentials and intrinsic motivation. The implicit behavioristic assumptions in many implementations (that badges will automatically motivate desired behaviors) overlook decades of research showing that extrinsic rewards can undermine intrinsic motivation when poorly designed. This is particularly concerning for climate education, where superficial engagement driven by credential-seeking could replace the deep, value-based commitment needed for sustained environmental action.

We must also confront an uncomfortable possibility raised by this review: that the very premise of using external recognition systems for climate education might be flawed. If the climate crisis demands fundamental transformation of consciousness and radical restructuring of our relationship with the environment, can this truly be fostered through systems of badges and micro-credentials? The complete absence of eco-pedagogical frameworks in digital recognition literature might not simply be a gap to address, but a signal that some forms of learning resist and should resist commodification into credentials. Future implementations must grapple with these motivational complexities, but also with the possibility that the most transformative aspects of climate education might need to occur outside formal recognition systems entirely.

Perhaps most significantly, our review reveals the complete absence of eco-pedagogical frameworks in digital credentialing—a gap that represents more than missing literature; it reflects a critical failure to connect recognition systems with the pedagogies most needed to address the climate crisis. Despite eco-pedagogy’s emphasis on critical consciousness, praxis, and transformative learning, no existing recognition systems incorporate these principles. This absence suggests that current systems may inadvertently perpetuate technocratic approaches to climate education while marginalizing the critical, action-oriented, and justice-focused dimensions essential for meaningful climate action.

These findings have important implications for the OpenPass4Climate project and the broader field of climate education. First, the project enters relatively uncharted territory, with the opportunity to pioneer frameworks that recognize not just what learners know about climate change, but how they critically engage with power structures, environmental injustices, and collective action for planetary sustainability. Second, the integration of eco-pedagogical principles into recognition systems remains an unrealized opportunity that could fundamentally reshape how we value and validate environmental learning.

We recommend that future recognition systems for climate education: (1) develop criteria that recognize praxis—the integration of critical reflection and environmental action; (2) create pathways validating community-based environmental engagement; (3) design assessment approaches that capture transformative learning journeys, not just outcomes; (4) establish quality assurance mechanisms that maintain rigor while allowing for contextual variation; and (5) build employer and societal understanding of the value of critical environmental consciousness alongside technical skills.

The scarcity of literature at the intersection of open recognition and climate education should not be seen as a limitation but as a call to action. As climate challenges intensify, educational systems must evolve to recognize and validate the full spectrum of competencies needed for planetary sustainability—from technical knowledge to critical consciousness, from individual skills to collective action capabilities. The OpenPass4Climate project has the potential to lead this transformation, creating recognition systems that honor the complexity and urgency of climate education in all its dimensions.

As detailed in our Future Research Directions section (Section 5), these priorities require sustained investigation by researchers, practitioners, and policymakers working at the intersection of open education and climate action.

This requires not taking recognition systems for granted as inherently positive developments, but rather critically examining their assumptions, limitations, and potential unintended consequences. Only through such critical engagement can we develop recognition frameworks that genuinely support the transformative learning needed to address the climate crisis.

## Ethics and consent

Ethical approval and consent were not required.

## Data Availability

No data are associated with this article.
