# Supplementary material for: Digital credentials for environmental competencies: A scoping review of open recognition systems in climate education
Source: Open Res Eur. 2026 Feb 11;5:138. Originally published 2025 May 19. [Version 4] doi: 10.12688/openreseurope.20048.4 (PMC12355165; doi:10.12688/openreseurope.20048.4)
Supplement: Peer review history

# Reviewer report: Reviewer response for version 3

**DOI:** 10.21956/openreseurope.22897.r54675

**Published:** 2026-01-27

**Authors:** Fahriye Altinay

**Affiliations:** 1 Near East University, Northern Cyprus, Turkey

Starting the introduction part of the article directly by OpenPass4Climate project limits scientific base of the article. It is better to point out general perspective of the article in relation to the aim of the research. In introduction, it is suggested to use update references and also each argumentation needs to be supported more references. For example, using one reference between two paragraphs isn't appropriate. Methodology section of the article is weak. There is a need to point out how reviews are analyzed. While making reviews, it is important to raise research questions. Future directions need to be added after presentation of findings.

Are all factual statements correct and adequately supported by citations?

Partly

Is the review written in accessible language?

Partly

Are the conclusions drawn appropriate in the context of the current research literature?

Partly

Is the topic of the review discussed comprehensively in the context of the current literature?

Partly

Reviewer Expertise:

educational technology, management, disability, sustainable development goals

I confirm that I have read this submission and believe that I have an appropriate level of expertise to state that I do not consider it to be of an acceptable scientific standard, for reasons outlined above.

# Response

**Published:** 2026-02-01

**Authors:** Pablo Martín-Ramos

**Affiliations:** Ingeniería Agrícola y Forestal, Universidad de Valladolid, Palencia, Castile and León, Spain

*We thank Dr. Altinay for taking the time to review our manuscript and for the constructive suggestions regarding the paper’s structure and methodological transparency. We have carefully considered each comment and implemented targeted improvements. Below we address each point in detail.*

**Reviewer comment 1**: “Starting the introduction part of the article directly by OpenPass4Climate project limits scientific base of the article. It is better to point out general perspective of the article in relation to the aim of the research.”

***Response***: *We thank the reviewer for this valuable observation. We agree that establishing the broader scientific and educational context before introducing the specific project strengthens the paper’s foundation and clarifies its contribution beyond the project itself.* *To address this, we have added a new framing paragraph at the very beginning of the Introduction that situates the review within the wider challenge of connecting open recognition systems with climate education. This paragraph establishes the general research problem—the underexplored intersection of digital credentials and climate education—before the OpenPass4Climate project subsection provides the applied context for our investigation.* *Change made: A new opening paragraph has been inserted at the beginning of the Introduction section, before the subsection “Overview of the OpenPass4Climate project.”*

**Reviewer comment 2:** “In introduction, it is suggested to use update references and also each argumentation needs to be supported more references. For example, using one reference between two paragraphs isn’t appropriate.”

***Response**: We appreciate the reviewer’s attention to citation density in the Introduction. We have strengthened the evidentiary base for key introductory arguments by adding supplementary citations from sources already included in our systematic review. Specifically, we have added supporting references (Zawacki-Richter et al., 2020; Wolz et al., 2021) alongside the existing citation to West & Cheng (2023) in the paragraph discussing the emergence of open badges and micro-credentials. This demonstrates that our claims are grounded in a broader body of literature.* *Regarding “updated references”, we must respectfully clarify an important methodological point. As stated in Section 2.5 (Limitations), this scoping review has a defined search window ending on January 31, 2025—an a priori boundary established following standard scoping review methodology (Arksey & O’Malley, 2005; Levac et al., 2010) to ensure transparency and reproducibility. Extending the search window during revision would reduce reproducibility and make the evidence base non-comparable with the search strategy reported in the Methods. We note that January 2025 is a recent cut-off, ensuring the review captures current literature within its defined scope.* *To clarify this for readers, we have added a brief statement in the Limitations section explaining that the search window was not extended during revisions.* *Changes made: (1) Additional citations added in Section 1.2 “The rise of open recognition systems”; (2) Clarifying statement added to Section 2.5 “Limitations”.*

**Reviewer comment 3**: “Methodology section of the article is weak. There is a need to point out how reviews are analyzed. While making reviews, it is important to raise research questions.”

***Response**: We thank the reviewer for encouraging greater methodological transparency. We acknowledge that while the current version provides detailed information about our scoping review methodology, including the Arksey & O’Malley (2005) and Levac et al. (2010) frameworks, databases searched, and inclusion/exclusion criteria, two aspects could be made more explicit: (1) the formulation of guiding research questions, and (2) the specific analytical procedure.* *To address this, we have made two additions: First, we have added three explicit Research Questions at the end of Section 2.1 (Research design). These are operationalized from our stated objectives in Section 1.4 and provide a clear investigative framework: - RQ1 addresses how open recognition systems are currently conceptualized and implemented; - RQ2 examines their application to climate education and eco-pedagogical activities; - RQ3 explores the challenges, tensions, and opportunities identified in the literature. Second, we have expanded the Data Analysis description in Section 2.4 to explain the thematic synthesis procedure, including the extraction matrix, inductive coding process, and how codes were grouped into the thematic categories that structure our Findings, Challenges, and Recommendations sections. These additions enhance transparency without altering the review’s substantive content.* *Changes made: (1) Three Research Questions inserted at the end of Section 2.1; (2) Data Analysis paragraph expanded in Section 2.4.*

**Reviewer comment 4**: “Future directions need to be added after presentation of findings.”

***Response***: *We agree that clearly articulated future directions are essential for readers. We would like to highlight that the current manuscript already includes a dedicated section titled “Future research directions” (Section 5), which appears after the Recommendations section and before the Conclusion. This section synthesizes specific avenues for future research drawn from the reviewed literature. Additionally, the Conclusion (Section 6) contains five numbered priorities for future recognition systems in climate education. However, we recognize that the framing of Section 5 may not have been sufficiently prominent or clearly linked to the preceding analysis. We have therefore revised its opening sentence to explicitly connect the future directions to our findings. We have also added a signposting reference in the Conclusion to ensure readers are directed to this content.* *Changes made: (1) Opening sentence of Section 5 “Future research directions” revised; (2) Cross-reference sentence added to the Conclusion.* *We trust these revisions address the reviewer’s concerns while maintaining methodological rigor and consistency with the substantial improvements already made in response to Reviewers 1 and 2. We appreciate the opportunity to strengthen the manuscript further.*

# Reviewer report: Reviewer response for version 3

**DOI:** 10.21956/openreseurope.22897.r58055

**Published:** 2025-08-14

**Authors:** Jouni Kekäle

**Affiliations:** 1 University of Eastern Finland, Joensuu, Finland

1. The corrections were good and the aspects I raised were taken well into consideration. I approve this happily.

Is the review written in accessible language?

Yes

Are the conclusions drawn appropriate in the context of the current research literature?

Yes

Is the topic of the review discussed comprehensively in the context of the current literature?

Yes

Reviewer Expertise:

NA

I confirm that I have read this submission and believe that I have an appropriate level of expertise to confirm that it is of an acceptable scientific standard.

# Reviewer report: Reviewer response for version 2

**DOI:** 10.21956/openreseurope.22473.r56102

**Published:** 2025-08-05

**Authors:** Jouni Kekäle

**Affiliations:** 1 University of Eastern Finland, Joensuu, Finland

**Your Report**

 The paper deals with open education recognition systems (RS) across formal, informal, and non-formal learning environments. The topic is broad as there are various learning environments, and RS is a developing category.  Particular attention is paid on micro-credentials, digital badges, and open educational resources in climate education. Research published between 2010 and 2024 were analyzed.

 Significant obstacles to RS adoption, include lack of standardization, limited awareness among employers, and technical and other implementation barriers. A practical aim is to “understand the mechanisms that facilitate the rapid replication of positive behaviors in environmental contexts” (introduction). The paper aligns with the OpenPass4Climate project which has aimed at portable pass to be utilized throughout one's life, designed to empower individuals from ages 10 to 80 to actively engage them in climate-related endeavor's and to take pride in the badges they earn.

 The aims are good, in common sense, as climate change is one of humanity’s grandest problems. However, the limitation of the RS approach, not discussed in the report, is that the approach studied appears to be based on a rather behavioristic understanding of human action, emphasizing reactions to external rewards. This circumvents the idea that humans may have different meanings to things, and they typically act in relation to their meanings and interpretations rather than to external technical stimuli directly, at least when there is no fundamental pressure involved. All people may not value such RS passes, nor take pride in them, while some people may do so, regardless of their attitude towards climate change mitigation.  According to many studies, extrinsic motivation and external rewards often tend to predict shallow cognitive engagement. Such problems and limitations are not considered here.

 The challenges of the approach studied include standardization and definitional issues, problems in a) institutional coherence and coordination, b) stakeholder awareness and acceptance, and technological and implementation barriers (technological readiness have been seen as a critical factor in micro-credential implementation). Some challenges are climate education specific.

 Recommendations are clear and straight forward. The paper concludes: “The success of micro-credentialing systems will depend on addressing challenges related to standardization, technological readiness, and stakeholder perceptions while maximizing opportunities for flexible, personalized learning pathways and enhanced employability in climate-related fields.”

 The report still does what it says: yet, discussions remain on a technical level, without contextualization, mainly by classifying different studies in the technical area. The handling remains on a rather superficial level. This is not to say that you should not or could not foster micro credentials or RS but the feeling remains that much of the approach appears to be taken for granted, and a bit more critical approach could get more out of the possibilities and problems of the approaches studied.

Is the review written in accessible language?

Yes

Are the conclusions drawn appropriate in the context of the current research literature?

Yes

Is the topic of the review discussed comprehensively in the context of the current literature?

Yes

Reviewer Expertise:

Doctor of psychology: 1997, 28 years ago 23 Years as human resources director at multidispilinary universities. Research background: higher education studies.

I confirm that I have read this submission and believe that I have an appropriate level of expertise to confirm that it is of an acceptable scientific standard, however I have significant reservations, as outlined above.

# Response

**Published:** 2025-08-07

**Authors:** Pablo Martín-Ramos

**Affiliations:** Ingeniería Agrícola y Forestal, Universidad de Valladolid, Palencia, Castile and León, Spain

*We thank the reviewer for the thoughtful and constructive feedback. The reviewer's expertise in higher education studies and work on HEIs' role in climate change mitigation provides valuable perspective for strengthening our manuscript. The concerns raised about theoretical grounding and critical depth have prompted important revisions. Below, we address each comment with specific modifications to the manuscript.*

**Reviewer comment 1**: “However, the limitation of the RS approach, not discussed in the report, is that the approach studied appears to be based on a rather behavioristic understanding of human action, emphasizing reactions to external rewards. This circumvents the idea that humans may have different meanings to things, and they typically act in relation to their meanings and interpretations rather than to external technical stimuli directly, at least when there is no fundamental pressure involved. All people may not value such RS passes, nor take pride in them, while some people may do so, regardless of their attitude towards climate change mitigation.  According to many studies, extrinsic motivation and external rewards often tend to predict shallow cognitive engagement. Such problems and limitations are not considered here.”

***Response**: The reviewer correctly identifies an important limitation in our initial analysis. The manuscript did not adequately address the theoretical assumptions underlying recognition systems, particularly regarding human motivation and meaning-making. The concern about behavioristic assumptions and the potential for external rewards to undermine deeper engagement is well-founded and supported by extensive research in motivational psychology. We have revised the manuscript to incorporate these theoretical considerations throughout, examining how recognition systems interact with intrinsic motivation and acknowledging that individuals construct different meanings from these credentials based on their values and contexts.* *Changes made:* *(1) New paragraph added to the Introduction section, after the paragraph ending "...explicit forms of recognition”* *(2) New paragraph added to section 3.1 "Badges and micro-credentials", after the paragraph discussing Currie et al. (2023) ending "...heightened learner fulfillment.”* *(3) Two new paragraphs added to section Section 3.4 "The eco-pedagogy gap", after the paragraph ending "...how we conceptualize and value environmental learning."* *(4) New section 4 - "Motivational and theoretical considerations", before current "Challenges" section, which becomes Section 5.* *(5) New subsection on “Addressing motivational complexity” added to the "Recommendations" section, before "Institutional integration and policy". The opening paragraph has of the section been updated accordingly.* *(6) Two new paragraphs added to the “Conclusion” section, after the paragraph discussing limited examples from Nordic tourism and climate adaptation.*  

**Reviewer comment 2**: "The report still does what it says: yet, discussions remain on a technical level, without contextualization, mainly by classifying different studies in the technical area. The handling remains on a rather superficial level. This is not to say that you should not or could not foster micro credentials or RS but the feeling remains that much of the approach appears to be taken for granted, and a bit more critical approach could get more out of the possibilities and problems of the approaches studied.”

***Response**: The reviewer correctly notes that the initial manuscript focused primarily on describing and classifying existing approaches without sufficient critical analysis. We have revised the manuscript to provide deeper engagement with underlying assumptions, tensions, and limitations of recognition systems. The additions include theoretical frameworks from motivational psychology and learning theory, critical examination of whose knowledge gets recognized, and explicit discussion of the tensions between standardization and meaningful learning.* *Changes made:* *(1) New paragraph added to section 3.3 "Recognition of prior and non-formal learning", after the final paragraph.* *(2) New paragraph added to section 3.2 "Open Educational Resources (OER) and Practices (OEP), MOOCs, and online learning", after the paragraph ending "...ecological issues."* *(3) New paragraph added at the end of Section 4.3 (in the new section on “Motivational and theoretical considerations”)* *(4) New paragraph added to the “Conclusion” section, after the sentence ending "...climate education in all its dimensions.".*

# Reviewer report: Reviewer response for version 2

**DOI:** 10.21956/openreseurope.22473.r56160

**Published:** 2025-07-16

**Authors:** Alexandra Okada

**Affiliations:** 1 Open University, Milton Keynes, UK

It is great to see all the items addressed. A final suggestion, which will increase the quality of the paper is the proof reading, references style and following the journal specifications.

Are all factual statements correct and adequately supported by citations?

Partly

Is the review written in accessible language?

Yes

Are the conclusions drawn appropriate in the context of the current research literature?

Partly

Is the topic of the review discussed comprehensively in the context of the current literature?

Partly

Reviewer Expertise:

Global Education,  Education for Sustainable Development, Open Learning and Digital Trasnformation

I confirm that I have read this submission and believe that I have an appropriate level of expertise to confirm that it is of an acceptable scientific standard.

# Reviewer report: Reviewer response for version 1

**DOI:** 10.21956/openreseurope.21685.r54673

**Published:** 2025-06-18

**Authors:** Alexandra Okada

**Affiliations:** 1 Open University, Milton Keynes, UK

The paper addresses a very interesting and relevant topic. Some key suggestions to enhance its rigor include making the Methodology and Findings sections more concise and providing a clearer reference to support the approach adopted.

**About the Abstract**

 The abstract presents a generally clear and well-structured overview of the paper. However, to enhance its coherence, precision, and overall impact, several areas could be improved with more concise and specific language.

 The abstract would benefit from explicitly clarifying the scope and nature of the literature review. Which method was used?

**Clarification of Open Educational Resources (OER) Recognition**

 The mention of OER in the context of recognition is somewhat vague. It is not clear whether (e.g. OER Participation, Completion, Certification?)  or other forms of recognition are being referenced. Please specify the exact nature of recognition related to OER that the paper addresses to avoid ambiguity.

**Specification of Challenges**

 The abstract references “challenges” but does so in general terms. More precise identification of which challenges are addressed is important for clarity. For instance, the terms “standardization,” “technological integration,” and “stakeholder collaboration” are broad and would benefit from further explanation or examples. Citing specific, well-defined challenges will strengthen the paper’s positioning and help readers grasp the precise obstacles in the field.

**Rationale Behind Findings**

 The abstract notes a growing importance and need for integration of learning recognition / qualifications (e.g. badges, credentials,) but the rationale or evidence supporting this claim is not sufficiently clear. It would be helpful to briefly indicate why this integration is important, for example by highlighting the impact, gap, or opportunity it addresses for learners and professional development on Climate Education.

**Focus of Conclusion**

 The conclusion, as described in the abstract, could be more sharply focused. Instead of mentioning promising practices summarise recommendations drawn and new directions or areas for further study.

 The abstract is key for providing stronger closure and a clear takeaway message aligned with the paper’s aims.

**About the paper**

 The methodology states that the criteria for the literature review: only publications in peer-reviewed journals. However, the final sample consists of a variety of content types: 69 articles, review papers, book chapters, and reports.  This must be revised for consistency.

 Please clarify your literature review approach—e.g., is it a narrative review, scoping review, or another type? While you mention peer-reviewed sources, your final sample includes diverse materials such as book chapters and reports. If the review is not fully systematic, please explain your selection rationale and method clearly.

 Would it be possible to include a table of results indicating journals (for articles), publishers (for book chapters), and funders (for research reports), along with the year and topic/field? Such a table, accompanied by a brief explanation, would be very helpful for readers to familiarize themselves with the literature before engaging with your findings.

 Considering the paper’s focus on climate education, significant findings related to this area could be made more visible and, if possible, extended to include more details about the learning outcomes and approaches for effective definition through micro-credentials, digital badging, or OER completion. For example:

 In this paragraph: “In climate education, open badges offer a promising way to acknowledge interdisciplinary skills essential for environmental action. Rollin (2021) demonstrates their use in higher education to assess transversal skills such as critical thinking and collaboration—key competencies for addressing climate challenges. This aligns with findings by West and Cheng (2023), who show how micro-credentials can support personalized learning pathways, motivating learners to develop sustainability skills through self-directed efforts.”

 Could you clarify how Rollin (2021) assesses transversal skills like critical thinking and collaboration and criteria used to provide open badges? How do West and Cheng (2023) support personalized learning pathways and their evaluation for recognition? Furthermore, how do Miller and Jorre de St Jorre (2024) exemplify micro-credentials demonstrating specific environmental competencies? Which competencies are emphasized, and what aspects do they highlight regarding standards and the missing contextual information about these credentials?

 There is significant information about Badges, OER and Microcredential; however, if the findings could focus more on climate education, the discussion and conclusion sections could be substantially enriched.

 The recommendations mention pedagogy, but this is not reflected in the findings. “Ecopedagogical activities”, which were a key part of the search, are not discussed in the findings, discussion, nor conclusion.

 Overall, the paper is highly relevant and topical. Adding these layers of specific, concise information would enable readers to better understand how research on the recognition of open learning—including digital badges, micro-credentials, and OER—underscores their potential to transform education and professional development, particularly in climate-related fields.

Are all factual statements correct and adequately supported by citations?

Partly

Is the review written in accessible language?

Yes

Are the conclusions drawn appropriate in the context of the current research literature?

Partly

Is the topic of the review discussed comprehensively in the context of the current literature?

Partly

Reviewer Expertise:

Global Education,  Education for Sustainable Development, Open Learning and Digital Trasnformation

I confirm that I have read this submission and believe that I have an appropriate level of expertise to state that I do not consider it to be of an acceptable scientific standard, for reasons outlined above.

# Response

**Published:** 2025-06-23

**Authors:** Pablo Martín-Ramos

**Affiliations:** Ingeniería Agrícola y Forestal, Universidad de Valladolid, Palencia, Castile and León, Spain

The paper addresses a very interesting and relevant topic. Some key suggestions to enhance its rigor include making the Methodology and Findings sections more concise and providing a clearer reference to support the approach adopted.

*Response:*

*Dear Dr. Okada,*

*Thank you for your thorough and constructive review. Your expertise in Global Education, Education for Sustainable Development, and Open Learning provides an invaluable perspective. We have taken your feedback as a directive for the substantial revisions that were required.*

*Your comments have been instrumental in helping us to re-evaluate our contribution. We now recognize that the most significant finding of our work is the critical gap between the burgeoning field of open recognition and its sparse, under-theorized application in climate education. Our revised manuscript has been restructured to frame this "evidence gap" as its central argument, allowing for a more incisive analysis of the challenges and opportunities for the field.*

*Below, we provide a point-by-point response detailing the comprehensive revisions we have undertaken. We have endeavored to be as specific as possible, providing the text that has been modified or added to the manuscript to ensure our revisions are transparent and directly address your concerns.*

**About the Abstract**

 The abstract presents a generally clear and well-structured overview of the paper. However, to enhance its coherence, precision, and overall impact, several areas could be improved with more concise and specific language.

*Response:*

*We agree that the abstract lacked precision and impact. It has been completely rewritten to address your points Q1-Q5 and to better reflect the revised paper's focus.*

**Q1.** The abstract would benefit from explicitly clarifying the scope and nature of the literature review. Which method was used?

*Response:*

*We agree that the method was not clearly stated. We have clarified that this is a scoping review with systematic search strategies.*

*The second sentence of the abstract has been replaced with: "This scoping review examines current practices in open recognition systems—including micro-credentials, digital badges, and open educational resources (OER) completion certificates and participation badges—with particular emphasis on their application and potential in climate education. Following systematic search strategies across eight databases, we analyzed 70 publications (2010-2024) comprising journal articles, book chapters, institutional reports, and conference proceedings to map existing evidence, identify key concepts, and uncover gaps in applying these systems to climate education."*

***Clarification of Open Educational Resources (OER) Recognition***

**Q2. **The mention of OER in the context of recognition is somewhat vague. It is not clear whether (e.g. OER Participation, Completion, Certification?)  or other forms of recognition are being referenced. Please specify the exact nature of recognition related to OER that the paper addresses to avoid ambiguity.

*Response:*

*As noted in the revision for Q1, we have specified the types of OER-related recognition discussed in the literature. In the abstract, we have replaced "open educational resources (OER)" with "open educational resources (OER) completion certificates and participation badges".*

***Specification of Challenges***

**Q3. **The abstract references “challenges” but does so in general terms. More precise identification of which challenges are addressed is important for clarity. For instance, the terms “standardization,” “technological integration,” and “stakeholder collaboration” are broad and would benefit from further explanation or examples. Citing specific, well-defined challenges will strengthen the paper’s positioning and help readers grasp the precise obstacles in the field.

*Response:*

*We accept this criticism. Our description of the challenges was too generic. We have specified the nature of each challenge category (without providing detailed examples that belong in the main text).*

*In the abstract, the challenges sentence has been replaced with: "The analysis reveals that while these systems have significant potential to bridge academic learning with professional demands and environmental action, their impact is limited by persistent challenges: lack of definitional consistency and quality-assurance standards across institutions; significant technological barriers to credential portability and verification; and lack of awareness and trust from employers, undermining these credentials’ currency in the labor market."*

***Rationale Behind Findings***

**Q4. **The abstract notes a growing importance and need for integration of learning recognition/qualifications (e.g. badges, credentials,) but the rationale or evidence supporting this claim is not sufficiently clear. It would be helpful to briefly indicate why this integration is important, for example by highlighting the impact, gap, or opportunity it addresses for learners and professional development on Climate Education.

*Response:*

*A concise rationale grounded in the specific demands of the climate and sustainability sectors has been included.*

*After the sentence about growing importance, we have added: "This integration is critical for validating the interdisciplinary and action-oriented skills demanded by the green economy, often acquired through the non-formal and informal learning pathways characteristic of environmental action and advocacy."*

***Focus of Conclusion***

**Q5.** The conclusion, as described in the abstract, could be more sharply focused. Instead of mentioning promising practices summarise recommendations drawn and new directions or areas for further study. The abstract is key for providing stronger closure and a clear takeaway message aligned with the paper’s aims.

*Response:*

*We agree that the conclusion of the abstract needs a stronger, synthesized message that moves beyond a simple summary of practices.*

*The original abstract’s conclusion has been replaced with a more impactful synthesis of our findings and their implications: “Our review concludes that a significant implementation and research gap exists between open recognition’s potential and its application in climate education. Most notably, we find a near-complete absence of recognition frameworks for eco-pedagogical activities, perpetuating focus on technical knowledge over transformative, action-oriented learning. We recommend that future work prioritize creating robust, co-designed assessment models for these critical competencies to bridge the gap between education and meaningful climate action.”*

**About the paper**

**Q6.** The methodology states that the criteria for the literature review: only publications in peer-reviewed journals. However, the final sample consists of a variety of content types: 69 articles, review papers, book chapters, and reports.  This must be revised for consistency.

*Response:*

*We acknowledge this inconsistency and have revised the methodology section to accurately reflect our inclusive approach.*

*In Section 2.2 "Selection criteria and process", we have replaced the bullet point "Published in peer-reviewed journals" with "Published in peer-reviewed journals, academic book chapters, institutional or governmental reports from recognized organizations, or peer-reviewed conference proceedings. This inclusive approach was adopted because much of the innovation in open recognition is driven by practice and documented in 'grey literature' that is crucial for a comprehensive understanding of implementation challenges and opportunities.”*

**Q7.** Please clarify your literature review approach—e.g., is it a narrative review, scoping review, or another type? While you mention peer-reviewed sources, your final sample includes diverse materials such as book chapters and reports. If the review is not fully systematic, please explain your selection rationale and method clearly.

*Response:*

*We have explicitly identified this as a scoping review and explained our approach.*

*In Section 2.1 "Research design", the first paragraph has been replaced with: "This study is a scoping review designed to map the existing literature on open recognition systems and their application to climate education. We chose this methodology, following the five-stage framework proposed by Arksey & O'Malley (2005) and further refined by Levac et al. (2010), as it is ideal for exploring emerging fields, clarifying key concepts, and identifying research gaps from a diverse body of evidence. Unlike a systematic review, which typically focuses on a narrow question and homogenous study types, a scoping review allows for the inclusion of varied literature, including the 'grey literature' essential for understanding this practice-driven field."*

*The two references have been added to the references list: *

- *Arksey, H., & O'Malley, L. (2005). Scoping studies: towards a methodological framework. International Journal of Social Research Methodology, 8(1), 19-32. https://doi.org/10.1080/1364557032000119616*
- *Levac, D., Colquhoun, H., & O'Brien, K. K. (2010). Scoping studies: advancing the methodology. Implementation Science, 5(1), 69. https://doi.org/10.1186/1748-5908-5-69*

**Q8.** Would it be possible to include a table of results indicating journals (for articles), publishers (for book chapters), and funders (for research reports), along with the year and topic/field? Such a table, accompanied by a brief explanation, would be very helpful for readers to familiarize themselves with the literature before engaging with your findings.

*Response:*

*A table summarizing the reviewed literature has been included - please see Table 1.*

*After Section 2.4 "Limitations", we have added a new paragraph indicating that "The distribution of our reviewed literature is summarized in Table 1. The predominance of general education sources (88.6%) compared to climate-specific publications (11.4%) reflects the current state of the field and reinforces our finding that the application of open recognition systems to climate education remains largely unexplored."*

*In addition, the first paragraph of Section 3 on "Current practices in open recognition systems" has been modified to reference the table: "As shown in Table 1, our analysis of 70 publications revealed extensive development in open recognition systems generally, but limited application to climate education specifically. Only eight publications (11.4%) directly addressed environmental or climate contexts, while the remainder discussed general educational applications. This disparity itself constitutes an important finding, suggesting that despite the urgent need for climate education and the affordances of digital recognition systems, their intersection remains largely unexplored. We present both the general developments and the limited climate-specific applications below, as understanding the broader landscape is essential for identifying opportunities for climate education integration."*

*Please note that a discrepancy between 69 (originally stated) and 70 (actual references, excluding the two indicated in previous comments, purely methodological) was detected and has been corrected in the “Screening and selection process” subsection.*

 Considering the paper’s focus on climate education, significant findings related to this area could be made more visible and, if possible, extended to include more details about the learning outcomes and approaches for effective definition through micro-credentials, digital badging, or OER completion.

 For example:

 In this paragraph: “In climate education, open badges offer a promising way to acknowledge interdisciplinary skills essential for environmental action. Rollin (2021) demonstrates their use in higher education to assess transversal skills such as critical thinking and collaboration, key competencies for addressing climate challenges. This aligns with findings by West and Cheng (2023), who show how micro-credentials can support personalized learning pathways, motivating learners to develop sustainability skills through self-directed efforts.”

**Q9.** Could you clarify how Rollin (2021) assesses transversal skills like critical thinking and collaboration and criteria used to provide open badges? How do West and Cheng (2023) support personalized learning pathways and their evaluation for recognition? Furthermore, how do Miller and Jorre de St Jorre (2024) exemplify micro-credentials demonstrating specific environmental competencies? Which competencies are emphasized, and what aspects do they highlight regarding standards and the missing contextual information about these credentials?

*Response:*

*We acknowledge that our initial description of these papers lacked specificity. Upon careful review, we found that while West and Cheng (2023) provide a valuable framework for personalized learning pathways through micro-credentials, and Miller and Jorre de St Jorre (2024) offer crucial insights into environmental employer perspectives, neither provides specific examples of climate education implementation. This reinforces our overall finding that the intersection of open recognition systems and climate education remains underdeveloped in the literature.*

*The current paragraph about climate education has been replaced with the following detailed, multi-paragraph analysis:*

*“In examining specific applications relevant to climate education, three studies offer insights, though only Miller and Jorre de St Jorre (2024) directly address environmental contexts. West and Cheng (2023) present a comprehensive framework for how open microcredentials support personalized learning across micro-, meso-, and macro-levels. At the micro-level, they identify four mechanisms through which badges support self-regulated learning: (1) helping learners set explicit goals through clear badge criteria, (2) establishing prerequisite pathways that scaffold complex learning, (3) connecting learning goals to professional development outcomes, and (4) providing both summative and formative feedback through badge metadata. They emphasize that microcredentials can "break content into small modular units, so learners can create their own learning pathways", with learners able to "hop on and off 'the formal education bus' when needed". While not specific to climate education, their framework suggests how environmental learning could be modularized into stackable credentials addressing different competencies. For example, a learner could earn badges for 'Carbon Footprint Analysis,' 'Stakeholder Communication,' and 'Policy Advocacy,' creating a personalized pathway towards a 'Climate Action Leadership' micro-credential that is both recognizable and meaningful.*

*The most directly relevant empirical study is from Miller and Jorre de St Jorre (2024), who examined employer perspectives on digital micro-credentials in environmental science through interviews with 22 environmental professionals in Australia. Their work provides clear evidence of strong employer demand for verifiable skills highly relevant to climate action, including:*

- *Communication and collaboration: the ability to "work collaboratively... [and] maintain a sense of where that sits in terms of the overall effort".*
- *Technical field skills: including environmental impact assessment, species identification, and data collection*
- *Systems thinking: the ability to understand "interconnections between social, economic, and environmental systems" *
- *Adaptability and lifelong learning: Employers emphasized the importance of "the want to keep learning and the ability to keep learning". *

*Crucially, while employers were enthusiastic, they demanded rigor and clarity, with one stating, "unless we're educated around what that symbol was actually around and the level of effort and rigour... then it doesn't have the obvious impact." This underscores the critical need for industry partnerships and transparent assessment standards for any climate-related credential.*

*Finally, Rollin (2021), though not focused on climate, offers a practical model for assessing the 'transversal skills' that are central to effective climate action. The study examines the 'Garantie Jeunes' program in France, which uses non-formal learning contexts to develop competencies like teamwork, autonomy, and responsibility in young adults (a full list of skills assessed is provided in the paper's appendix, Table A1). While the paper does not offer detailed assessment rubrics, it highlights a methodology for recognizing skills developed through practical experience and structured reflection. This approach serves as a direct model for recognizing skills gained through community-based climate projects, advocacy, or volunteerism—activities that are rich in learning but often remain un-credentialed.”*

*The challenges section under "Stakeholder awareness and acceptance" has also been modified to include the following information:*

*“Miller and Jorre de St Jorre's (2024) findings reinforce these challenges in the environmental sector specifically. They found that environmental employers often relied on proxy indicators like work experience and internships because they lacked familiarity with digital credentials. One employer admitted: "trying to get a feel for the person and their skillsets beyond the CV is part of the challenge". This suggests that even in fields directly relevant to climate action, significant education and trust-building are needed before micro-credentials can fulfill their potential.”*

*In addition, in the recommendations under "Employer and stakeholder alignment", we have also included:*

*“Building on Miller and Jorre de St Jorre's (2024) environmental sector findings, partnerships should: *

- *Include employers in credential design to ensure relevance to workplace needs *
- *Provide clear metadata about assessment methods and standards*
- *Balance comprehensive evidence with reviewability constraints*
- *Emphasize industry endorsement to build trust*
- *Create mechanisms for employers to quickly understand credential value without an extensive portfolio review.”*

**Q10.** There is significant information about Badges, OER, and Microcredential; however, if the findings could focus more on climate education, the discussion and conclusion sections could be substantially enriched.

*Response:*

*We appreciate your observation about the imbalance between general and climate-specific content. We acknowledge that climate education applications are underrepresented in our findings. This actually reflects the state of the literature - only 11.4% of sources addressed environmental/climate contexts. Rather than artificially inflate climate connections where they do not exist in the literature, we have chosen to be transparent about this gap and analyze its implications. We believe this approach (acknowledging the limited intersection as a finding itself) provides more value than overstating tenuous connections. The enriched conclusion now frames this gap as both a challenge and an opportunity for advancing climate education through digital recognition systems.*

*We have modified the beginning of Section 3 "Current practices in open recognition systems" (replacing the current first sentence), as discussed in the response to query 8 above.*

*The Conclusion has already been substantially revised to address this comment through: *

- *Explicit acknowledgment of the limited climate focus as a finding *
- *Analysis of the eco-pedagogy gap *
- *Framing the limitation as an opportunity for OpenPass4Climate *
- *Specific recommendations for climate education applications*
- *A call to action for developing recognition systems for "planetary sustainability"*

*Throughout Sections 3.1-3.5, we have now: *

- *Added specific details from Miller & Jorre de St Jorre (2024) on environmental employers *
- *Created an entire section (3.5) on the eco-pedagogy gap *
- *Integrated climate-relevant examples where they exist *
- *Been transparent about where connections are theoretical rather than empirical*

**Q11.** The recommendations mention pedagogy, but this is not reflected in the findings. “Ecopedagogical activities”, which were a key part of the search, are not discussed in the findings, discussion, nor conclusion.

*Response:*

*We acknowledge that climate education applications are underrepresented in our findings, which actually reflects the state of the literature. A new subsection has been added to Section 3 "Current practices in open recognition systems":*

*“The Ecopedagogy Gap: A Critical Absence in Digital Recognition*

*Our search for literature combining 'ecopedagogical activities' with open recognition systems yielded zero results, despite ecopedagogy's growing importance in climate education. This absence is particularly striking given the theoretical alignment between eco-pedagogical principles and the affordances of digital credentials.*

*Eco-pedagogy, rooted in Paulo Freire's critical pedagogy (Freire, 1970), aims to develop "planetary consciousness" and foster social and environmental justice through transformative educational practices (Gadotti, 2008; Kahn, 2010). Its methods include experiential learning, place-based education, systems thinking workshops, and action-oriented climate projects (Hung, 2021; Kopnina, 2020; Misiaszek, 2020). These activities emphasize critical consciousness, praxis (reflection and action), and transformative learning—elements that could be recognized through carefully designed digital credentials.*

*The complete absence of eco-pedagogical frameworks in the digital recognition literature suggests several profound barriers: a philosophical tension between the standardization required for badges and the contextual, transformative nature of eco-pedagogy (Andreotti, 2006); the inherent difficulty in assessing outcomes like 'critical consciousness' and ‘planetary citizenship’ (Misiaszek, 2018); the privileging of technical competencies over the critical, values-based learning central to eco-pedagogy in current badging systems (Kopnina, 2020); and a likely disconnect between the educational technology and critical pedagogy communities.*

*This gap has significant implications. While institutions implement diverse eco-pedagogical activities—from climate action workshops to community-based environmental projects—these transformative learning experiences remain unrecognized in formal credentialing systems. This invisibility may perpetuate the dominance of technical approaches to climate education over critical, action-oriented pedagogies.*

*The absence of eco-pedagogical approaches in digital recognition systems represents more than a technical gap—it reflects deeper tensions in how we conceptualize and value environmental learning. As Misiaszek (2018) argues, eco-pedagogy is "essentially literacy education for reading and re-reading human acts of environmental violence." Such critical analysis resists easy codification into badge criteria. Yet this challenge also presents an opportunity. Digital recognition systems could evolve beyond competency verification to recognize transformative learning processes through badges that document critical reflection on environmental justice issues, credentials recognizing sustained community environmental action, portfolios capturing the development of "planetary citizenship" (Gadotti, 2008), and recognition for facilitating others' environmental conscientization. The OpenPass4Climate project is uniquely positioned to bridge this gap by developing recognition frameworks that honor both the measurable and transformative aspects of climate education.”*

*In addition, we have added to the recommendations section, under "Pedagogical approaches", the following paragraph:*

*“Ecopedagogical Integration To bridge the identified gap, badge design for climate education should be guided by eco-pedagogical principles: *

- *Develop criteria that recognize praxis—the integration of critical reflection and environmental action (Freire, 1970) *
- *Create pathways validating community-based environmental engagement and collective action, not just individual achievement. *
- *Design e-portfolios that capture transformative learning journeys, not just static outcomes *
- *Include peer recognition mechanisms reflecting eco-pedagogy's emphasis on horizontal learning relationships. *
- *Integrate place-based learning achievements that connect global climate challenges to local contexts (Sobel, 2004).” *

*The Conclusion section has been updated accordingly:*

*“Perhaps most significantly, our review reveals the complete absence of ecopedagogical frameworks in digital credentialing—a gap that represents more than missing literature; it reflects a critical failure to connect recognition systems with the pedagogies most needed to address the climate crisis. Despite ecopedagogy's emphasis on critical consciousness, praxis, and transformative learning, no existing recognition systems incorporate these principles. This absence suggests that current systems may inadvertently perpetuate technocratic approaches to climate education while marginalizing the critical, action-oriented, and justice-focused dimensions essential for meaningful climate action.”*

*As for the references, nine new references have been included: *

- *Andreotti, V. (2006). Soft versus critical global citizenship education. Policy & Practice: A Development Education Review, 3, 40-51. Available at: https://www.developmenteducationreview.com/issue/issue-3/soft-versus-critical-global-citizenship-education *
- *Freire, P. (1970). Pedagogy of the oppressed. Continuum. New York, NY, USA. Available at: https://files.libcom.org/files/Paulo%20Freire,%20Myra%20Bergman%20Ramos,%20Donaldo%20Macedo%20-%20Pedagogy%20of%20the%20Oppressed,%2030th%20Anniversary%20Edition%20(2000,%20Bloomsbury%20Academic).pdf *
- *Gadotti, M. (2008). Education for sustainability: A contribution to the decade of education for sustainable development. International Review of Education, 54(5-6), 623-635. https://doi.org/10.5585/eccos.n54.16138 *
- *Hung, R. (2021). Ecopedagogy and education. In Oxford Research Encyclopedia of Education. Oxford University Press. https://doi.org/10.1093/acrefore/9780190264093.013.1502 *
- *Kahn, R. (2010). Critical pedagogy, ecoliteracy, and planetary crisis: The ecopedagogy movement. Peter Lang Pub Inc, New York, NY, USA. *
- *Kopnina, H. (2020). Education for the future? Critical evaluation of education for sustainable development goals. The Journal of Environmental Education, 51(4), 280-291. https://doi.org/10.1080/00958964.2019.1710444 *
- *Misiaszek, G. W. (2018). Educating the global environmental citizen: Understanding ecopedagogy in local and global contexts. Routledge, New York, NY, USA. *
- *Misiaszek, G. W. (2020). Ecopedagogy: Teaching critical literacies of 'development', 'sustainability', and 'sustainable development'. Teaching in Higher Education, 25(5), 615-632. https://doi.org/10.1080/13562517.2019.1586668 *
- *Sobel, D. (2004). Place-based education: Connecting classrooms & communities. The Orion Society, Great Barrington, MA, USA. *

Overall, the paper is highly relevant and topical. Adding these layers of specific, concise information would enable readers to better understand how research on the recognition of open learning—including digital badges, micro-credentials, and OER—underscores their potential to transform education and professional development, particularly in climate-related fields.

*Response:*

*We thank the reviewer for these detailed comments, which have helped us identify significant weaknesses in our manuscript. We are confident that these substantial revisions will address your concerns and transform the manuscript into a stronger, more rigorous, and more impactful contribution. Thank you once again for your time, your expertise, and your feedback. Sincerely,*

*​​​​​​​Pablo Martín-Ramos and co-authors*
